# Regulation of Peripheral Inflammation by a Non-Viable, Non-Colonizing Strain of Commensal Bacteria

**DOI:** 10.3389/fimmu.2022.768076

**Published:** 2022-02-02

**Authors:** Kritika Ramani, Taylor Cormack, Adam N. R. Cartwright, Aula Alami, Pooja Parameswaran, Marynawal Abdou, Iris Wang, Kristie Hilliard-Barth, Shannon Argueta, Divya Raghunathan, Will Caffry, Christopher J. H. Davitt, Fabian B. Romano, Aylwin Ng, Valeria Kravitz, Tyler Rommel, Maria Sizova, Esra Uckun Kiran, Pallavi Pradeep, Holly E. Ponichtera, Tanmoy Ganguly, Mark Bodmer, Andrea Itano

**Affiliations:** Evelo Biosciences, Cambridge, MA, United States

**Keywords:** Th1, Th17, Th2, inflammation, CNS, bacteria, gastrointestinal tract

## Abstract

The gastrointestinal tract represents one of the largest body surfaces that is exposed to the outside world. It is the only mucosal surface that is required to simultaneously recognize and defend against pathogens, while allowing nutrients containing foreign antigens to be tolerated and absorbed. It differentiates between these foreign substances through a complex system of pattern recognition receptors expressed on the surface of the intestinal epithelial cells as well as the underlying immune cells. These immune cells actively sample and evaluate microbes and other particles that pass through the lumen of the gut. This local sensing system is part of a broader distributed signaling system that is connected to the rest of the body through the enteric nervous system, the immune system, and the metabolic system. While local tissue homeostasis is maintained by commensal bacteria that colonize the gut, colonization itself may not be required for the activation of distributed signaling networks that can result in modulation of peripheral inflammation. Herein, we describe the ability of a gut-restricted strain of commensal bacteria to drive systemic anti-inflammatory effects in a manner that does not rely upon its ability to colonize the gastrointestinal tract or alter the mucosal microbiome. Orally administered EDP1867, a gamma-irradiated strain of *Veillonella parvula*, rapidly transits through the murine gut without colonization or alteration of the background microbiome flora. In murine models of inflammatory disease including delayed-type hypersensitivity (DTH), atopic dermatitis, psoriasis, and experimental autoimmune encephalomyelitis (EAE), treatment with EDP1867 resulted in significant reduction in inflammation and immunopathology. *Ex vivo* cytokine analyses revealed that EDP1867 treatment diminished production of pro-inflammatory cytokines involved in inflammatory cascades. Furthermore, blockade of lymphocyte migration to the gut-associated lymphoid tissues impaired the ability of EDP1867 to resolve peripheral inflammation, supporting the hypothesis that circulating immune cells are responsible for promulgating the signals from the gut to peripheral tissues. Finally, we show that adoptively transferred T cells from EDP1867-treated mice inhibit inflammation induced in recipient mice. These results demonstrate that an orally-delivered, non-viable strain of commensal bacteria can mediate potent anti-inflammatory effects in peripheral tissues through transient occupancy of the gastrointestinal tract, and support the development of non-living bacterial strains for therapeutic applications.

## Introduction

The small intestine is a central hub and regulator of systemic immunity. It acts as a sensory window to the external world: relaying messages from the gut contents to peripheral tissues, based on what it sees passing through. The mucosal immune system is a complex cellular network that acts to govern peripheral responses to diverse challenges. Multicellular organisms exist as holobionts, comprising. The macroscopic host and symbiotic commensal microbes. Microbes in the gut engage in dialogue with host cells like dendritic cells, macrophages, and epithelial cells to promote, calibrate and terminate immune responses in the most appropriate manner to impact systemic inflammation (1). One of the primary modes of dialogue between microbes and host cells is through recognition of microbial associated molecular patterns. Pattern recognition receptors like toll like receptors (TLR) respond to microbial ligands to drive numerous signals that are transmitted throughout the body by the circulation of cells, neurological signals, and hormones. Microbes can induce tolerogenic signals in the gut mesenteric lymph nodes and Peyer’s patches to produce cytokines and chemokines that drive immunomodulatory functions (2). TLR engagement can also result in conditioning of gut epithelial cells or lead to production of regulatory cytokines such as IL-10 (3). At the cellular level, immune protective responses include induction of gut specific subsets of CD4^+^ Foxp3^+^ regulatory T cells locally in the gut as well as at the site of inflammation (4, 5). Intestinal Tregs control aberrant immune responses in a context dependent manner to produce IL-10 and regulate proinflammatory cell subsets by regulating availability of growth factors (6). Additionally, there is evidence for production of gut CD103^+^ DCs and tolerogenic macrophages that lead to reduction of proinflammatory Th1 and Th17 cells (7) that can have anti-inflammatory effects.

Several reports have highlighted a role for microbes in mitigating inflammation in preclinical models of autoimmune diseases like multiple sclerosis and arthritis as well as driving anti-tumor responses in cancer models (7–9). Perturbations in beneficial microbe niches have been correlated with increased inflammation in various diseased states (10–13). This has led to a plethora of current approaches that focus on correcting the dysbiosis associated with diseases using fecal microbiome transplants or consortia of bacteria to recolonize lost niches. While there have been some successes, these methods have often been marred by their lack of specificity and raise the concern of donor variability and quality control that could pose a serious challenge to developing safe therapies.

Here, we describe a distinct and unique approach of oral treatment with a non-living microbe to drive systemic anti-inflammatory effects. By screening human commensal microbes for their ability to modulate systemic inflammation upon oral dosing, we have identified a particular microbial strain from the *Veillonella* genus with specific pharmacological properties. *Veillonella* are found in several niches of the human body including mouth, lungs, gastrointestinal and genitourinary tracts, and are a normal component of a healthy human microbiome (14, 15). Recent data suggest that *Veillonella* may have a protective role and aid in early childhood immune system development. Epidemiological studies from infants have demonstrated that presence of *Veillonella* is negatively correlated with asthma (11), and bronchiolitis (13). *Veillonella* have also been reported in abundance in high performing athletes due to high lactate generation during exercise and tied to improved physical performance in mice that were treated with *Veillonella* (16).

In this report, we demonstrate that EDP1867 has a wide range of anti-inflammatory effects *in vitro* and *in vivo*, and we discuss mechanisms of inflammation resolution mediated through the mucosal immune system.

## Results

### Small Intestine Derived Bacterial Strain *Veillonella parvula* Induces IL-10 *In Vitro* and Resolves Inflammation *In Vivo*

Our central premise is that single strains of bacteria isolated from human small intestinal tissues can have robust anti-inflammatory properties and can therefore be developed as therapeutics with well-defined pharmacological activities. We isolated a strain of *Veillonella parvula (V. parvula*) from a fresh ileostomy sample of an IBD patient in remission. *V. parvula* is a gram-negative, obligate anaerobe that belongs to the Negativicutes class and is a natural commensal found in the oral cavity, gastrointestinal and genitourinary tracts. We developed a non-viable form of *V. parvula* by gamma irradiating the live microbe (designated as EDP1867).

**Figure 1:**
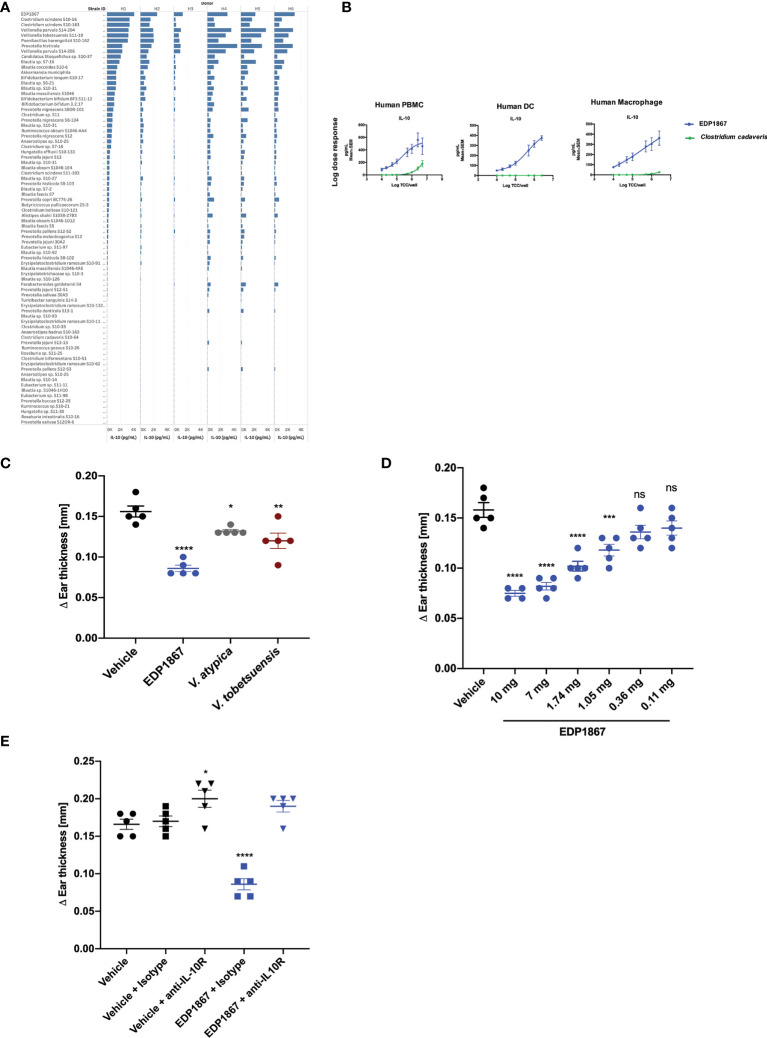
EDP1867 induces IL-10 *in vitro* and resolves inflammation *in vivo*. **(A)** Human Macrophages were stimulated with different anaerobic bacterial strains for 24h and flushed with 1% oxygen and supernatants were collected to test for cytokine levels by MSD. Data shown represent collective data from 6 independent human donors. **(B)** Human PBMCs, Dendritic Cells and Macrophages were stimulated with EDP1867 bacterial strains for 24h and flushed with 1% O_2_ and supernatants were collected to test for cytokine levels by MSD. Data shown represent collective data from 6 independent human donors. **(C)** DTH response to KLH. C57BL/6 mice were immunized with KLH and CFA on day 0 s.c. and challenged in the ear i.d. 9 days later with KLH. Mice were orally dosed daily from the day after immunization through ear challenge with vehicle or EDP1867; TCC-2.84E+10. Ear inflammation was measured on day 9. Change in ear thickness (n = 5 mice/group) for groups dosed with EDP1867; TCC -7.8E+11 and other non-replicating *Veillonella* strains (TCC-3.8E+11- 1.03E+12). **(D)** DTH was performed as previously described. Mice were orally dosed daily from the day after immunization through ear challenge with vehicle or EDP1867 (TCC 2.16E+12). Ear inflammation was measured on day 9. Change in ear thickness (n = 5 mice/group) demonstrate dose dependent effects of EDP1867. **(E)** EDP1867 acts through IL-10R pathway to reduce ear inflammation. Mice in various groups were treated with IL-10R blocking antibody on days 2,4, and 6 as indicated (EDP1867 TCC 2.16E+12). Representative figure from n = 2 experiments with 5mice/group in each experiment; All data show mean ± SEM. *p < 0.05, **p < 0.01, ***p < 0.001, ****p < 0.0001, ns, not significant. as determined by unpaired Student’s t-test. DTH, delayed-type hypersensitivity; KLH, keyhole limpet hemocyanin; CFA, complete Freund’s adjuvant; TCC, total cell count; s.c., subcutaneous; i.p., intraperitoneal; i.d., intradermal.

In order to compare the ability of different commensal bacterial strains, including EDP1867, to induce the anti-inflammatory cytokine IL-10 from primary human immune cells, fresh primary human CD11b^+^ immune cells were purified from the peripheral blood mononuclear cells (PBMC) of 6 healthy human donors and individually co-cultured with 87 unique bacterial strains representative of human microbiome that were isolated from numerous human fecal and oral samples, or probiotic mixtures. These strains were irradiated to be comparable to EDP1867 (an irradiated strain of *Veillonella parvula*). The bacterial screening panel included representative species from 17 taxonomic families and 25 genera that belong to phyla Actinobacteria, Firmicutes, Proteobacteria, and Bacteroidetes. The data show that of the >80 unique commensal strains evaluated, increased levels of IL-10 were induced from all donors in response to stimulation with EDP1867 compared with several closely related species (**Figure 1A** and **Supplementary Figure 1A**). In an *in vitro* setting mimicking an inflamed cell environment, EDP1867 was one of the highest inducers of IL-10 in comparison with other gamma irradiated *Veillonella* species (**Supplementary Figure 1B**). EDP1867 did not induce high levels of pro-inflammatory cytokines IP-10 or TNFa compared to other commensal anaerobic strains (**Supplementary Figure 1C**). Furthermore, based on the results from a single dose, a dose-response of EDP1867 was performed using isolated human PBMCs, DCs, and macrophages from whole blood, which were cultured with a range of doses from 10^4^ – 5 x 10^6^ total cell count (TCC) for 24 hours. We compared the dose-response of EDP1867 to that of one of the strains that had induced the least amount of IL-10, *Clostridium cadaveris* to confirm that a high and low inducer of IL-10 would continue to show differences across a range of doses. A dose-dependent production of IL-10 from PBMCs, DCs and macrophages was observed, whereas the strain of *Clostridium cadaveris* induced only a relatively low amount of IL-10 from PBMCs at the highest doses, and induced little or no IL-10 from DCs or macrophages, even at the highest dose. These results demonstrate pharmacological activity of EDP1867 on immune cells, and establish EC50 of 5.2 log TCC for PBMCs, 4.8 log TCC for DCs, and 4.9 log TCC for macrophages (**Figure 1B**).

To determine efficacy of EDP1867 as a therapeutic agent *in vivo*, we tested it in a DTH mouse model. Mice were sensitized by subcutaneous injection with keyhole limpet hemocyanin (KLH) emulsified with Complete Freund’s Adjuvant (CFA). Eight days after sensitization, the mice were challenged by intradermal ear injection with KLH. DTH response was evaluated 24 hours post-challenge-, which represents the peak of disease in this model. Mice were orally dosed daily for 8 days. EDP1867 was the most efficacious strain in lowering ear inflammation compared to other closely related *Veillonella* species (**Figure 1C**) and displayed dose dependent efficacy (**Figure 1D**). The anti-inflammatory effect of EDP1867 *in vivo* was evaluated by blocking the IL-10 signaling pathway during DTH. Co-administration with anti-IL-10R antibody impaired the effect of EDP1867 in resolution of ear inflammation suggesting that EDP1867 exerts its anti-inflammatory activity in part through the IL-10 pathway (**Figure 1E**).

Taken together, a non-viable form of *V. parvula*, EDP1867 demonstrates effectiveness as an anti-inflammatory therapeutic agent.

### Orally Administered EDP1867 Is Gut Restricted and Transits Rapidly Through the Intestine Without Alteration of the Gut Microbial Flora

**Figure 2:**
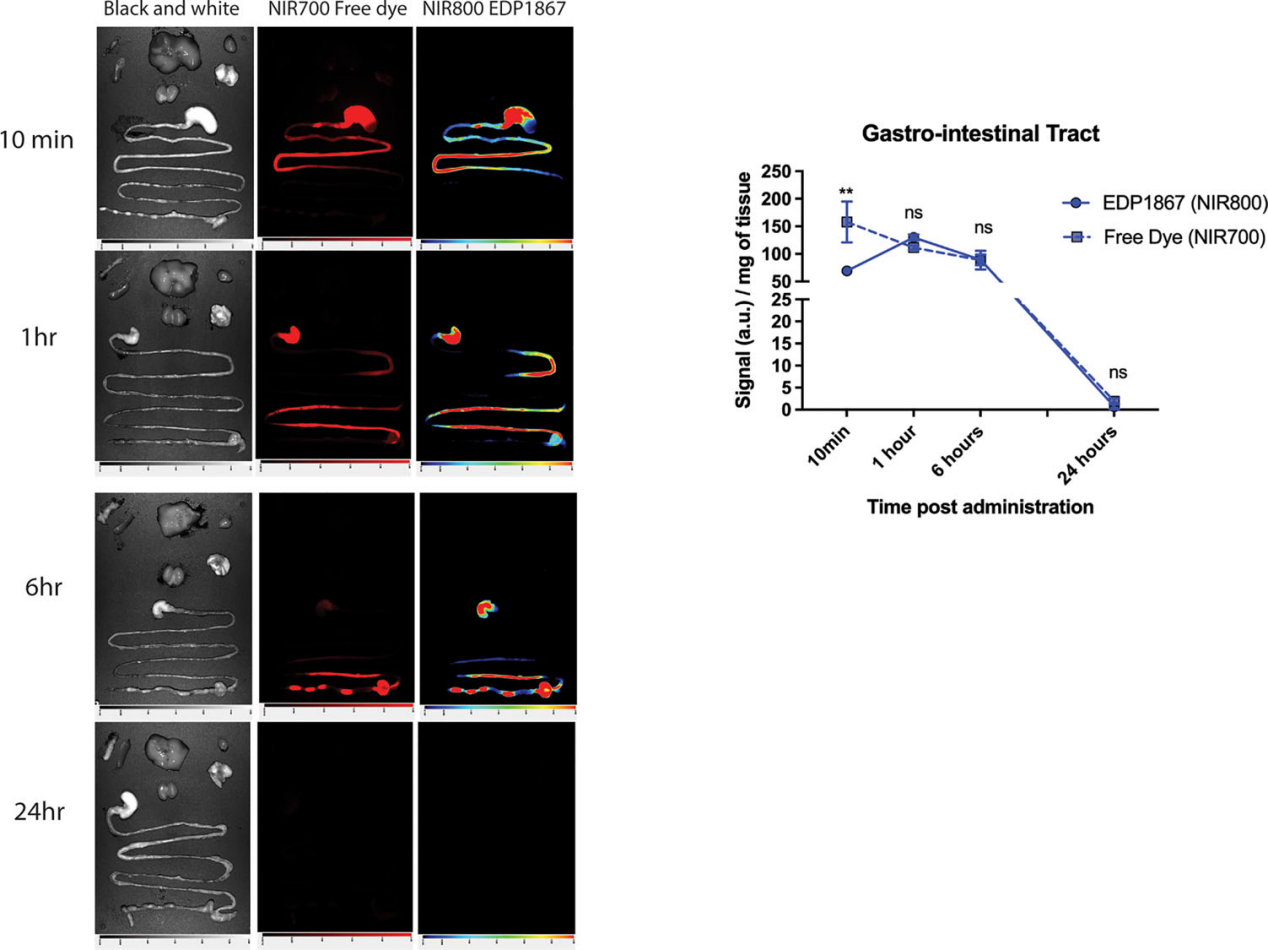
EDP1867 is gut restricted and transits through GI tract within 24hrs. Biodistribution of EDP1867. Following a single oral administration of EDP1867 (TCC – 1E+9), exposure of the small intestine occurs by 10 mins and complete exposure in 1 hour. By 6 hours, EDP1867 is incorporated into fecal pellets and by 24 hours signal in GIT diminishes suggesting complete excretion of the microbe with no detectable persistence. No systemic exposure is observed at any timepoint and >99% of the total signal remains in the GIT. Left column– brightfield images, middle- free dye, right- EDP1867 overlapped with free dye GIT- gastrointestinal tract. n = 3 mice/timepoint/group. Data are represented as mean + SEM. p values are represented as **p < 0.01, ns > 0.1, as determined by 2-way ANOVA followed by Sidak’s multiple comparison test.

EDP1867 is an orally delivered, gut restricted agent that exerts its pharmacological effects on the mucosal immune system. A study was designed to determine the biodistribution of EDP1867 in a mouse following oral treatment and tracked transit through the gastrointestinal (GI) tract. Oral administration of EDP1867 led to a transient rise in the GI tract. Complete exposure of the small intestine occurred within 1 hour post oral administration of EDP1867. EDP1867 was only detected in the intestine and stool for up to 12 hours post-treatment after a single dose (**Figure 2**). Importantly, EDP1867 was not detected outside the GI tract at any time point above background fluorescence as determined by the free dye control (**Supplementary Figure 2**). Since EDP1867 is non-viable, lack of persistence in the GI tract suggests that it does not require colonization to achieve its pharmacological activity. In an experiment to determine the effects of EDP1867 on the microbiome *in vivo, we c*onfirmed that there were no discernible changes to the microbiome composition at various time points after dosing (**Supplementary Figure 3**).

Together, these data demonstrate that EDP1867 is luminally restricted with undetectable systemic exposure following oral dosing and does not alter the microbiome.

### EDP1867 Inhibits Cutaneous Inflammation in Imiquimod Driven Model of Psoriasis

**Figure 3:**
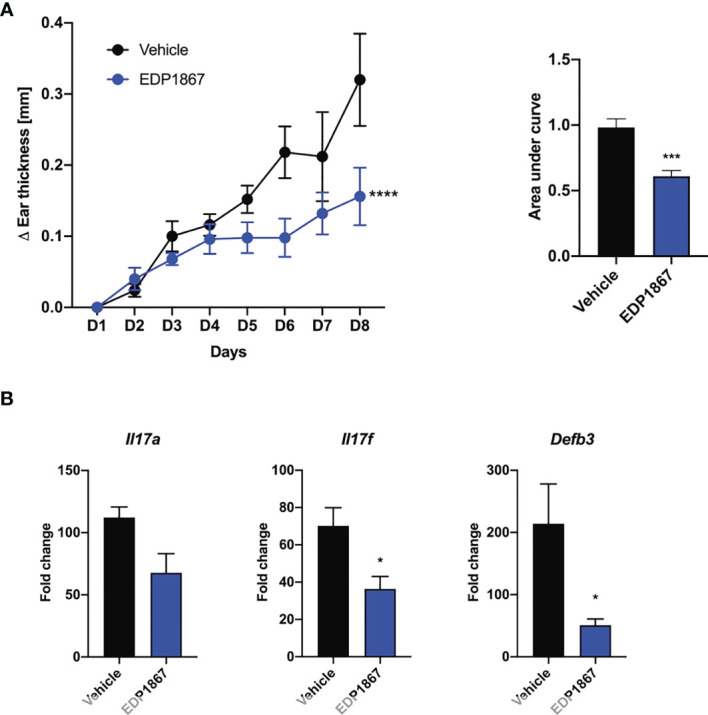
EDP1867 alleviates skin inflammation in imiquimod-induced psoriasis. Imiquimod induced psoriasis. BALB/c mice were topically treated with 20mg 5% imiquimod for 7 days on the ear. Mice were orally dosed daily from day 1 through 7 with vehicle or EDP1867 (TCC – 7.8E+11). **(A)** Ear inflammation over the course of 7 days and area under the curve. **(B)** mRNA transcript levels for *Il17*a, *Il17f* and *Defb3* measured by qPCR. Data are representative from 2 experiments with n = 5/group. All data show mean ± SEM. *p < 0.05, ***p < 0.0005, ****p < 0.0001, as determined by unpaired Student’s t-test.

Efficacy in a T cell mediated model like DTH suggested an anti-inflammatory role for EDP1867. We wanted to investigate the efficacy of EDP1867 in disease models with a strong T cell component. We chose the imiquimod-induced psoriasis model where Th17 cell associated IL-23/IL-17 cytokine axis plays a pivotal role. This model recapitulates aspects of human disease, including clinical and histological characteristics like human psoriasis, such as epidermal thickening, scaling and erythema, as well as infiltrates of T cells, neutrophils, and dendritic cells (17). Mice were sensitized on the ear with imiquimod cream daily for 7 consecutive days and orally dosed daily with EDP1867. EDP1867 treated mice showed substantially lower ear inflammation over the course of disease progression (**Figure 3A**). At termination of study, transcript levels in the ear tissue revealed reduction in *Il17a, Il17f and Defb3* levels upon treatment with EDP1867 in comparison to vehicle (**Figure 3B**).

### EDP1867 Ameliorates Inflammation in Murine Model of Relapsing-Remitting Multiple Sclerosis

**Figure 4:**
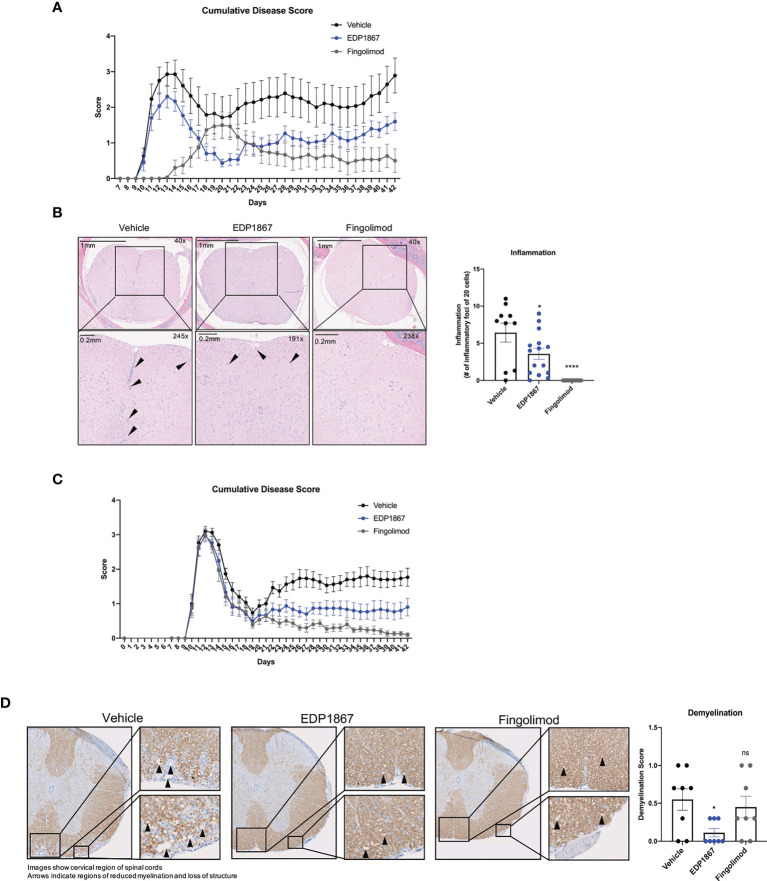
EDP1867 displays efficacy treating neuroinflammation in a model for relapsing remitting MS. PLP driven EAE. EAE was induced in SJL mice by immunization with PLP 139-151 in CFA on day 0, hour 0 and PTX was administered on day 0, hour 2. **(A)** Prophylactic dosing – vehicle, EDP1867 (TCC- 8.46E+10) and fingolimod (1mg/kg) were orally dosed daily for 41 days. Cumulative EAE scores of mice. Clinical scores were assessed daily for the duration of the experiment. ****p ≤ 0.00005 by Unpaired t-test with Welch’s correction was used to calculate p-value in cumulative AUC for the EAE score. **(B)** Representative hematoxylin and eosin (H&E)-stained images of the brain of spinal cords of mice treated with EDP1867, Vehicle or Fingolimod. Spinal cord sections are enlarged at 289X magnification to show regions with inflammation and inflammatory loci. Data for **Figures 5B, C** are representative of 2 independent experiments (n = 15 mice per group). **(C)** Therapeutic dosing – vehicle, EDP1867 (TCC- 8.46E+10) and fingolimod (1mg/kg) were orally dosed from day 10 to day 41. Cumulative EAE scores of mice. Clinical scores were assessed daily for the duration of the experiment. ****p ≤ 0.00005 by Unpaired t-test with Welch’s correction was used to calculate p-value in cumulative AUC for the EAE score. **(D)** Representative Luxol blue-stained images of the brain of spinal cords of mice treated with EDP1867, Vehicle or Fingolimod. Data for **Figures 5D, E** are representative from n = 15 mice per group. *p ≤ 0.05 by unpaired Student’s t-test. PLP, proteolipid protein; EAE, experimental autoimmune encephalomyelitis; PTX, pertussis toxin; CFA, complete Freund’s adjuvant. ns, not significant.

We also investigated the ability of EDP1867 to alleviate a second more chronic Th17-driven disease, by assessing its effects in a murine experimental autoimmune encephalomyelitis (EAE) model of relapsing remitting multiple sclerosis (MS). EAE, a widely used model of demyelinating diseases, is induced by immunization with a spinal cord antigen, proteolipid protein (PLP), a major protein component of CNS myelin. PLP injected in mice together with pertussis toxin disrupts the blood brain barrier, leading to remitting and relapsing demyelinating disease. Mice were scored daily for disease onset and progression as per the standard EAE scoring criteria, reflecting the degree of motor deficit. Mice began to display motor impairment by day 9 and reached peak clinical disease scores by day 15. In a prophylactic treatment regimen where dosing started on day 1, EDP1867 showed significant reduction in clinical score compared to vehicle treated animals over the course of the disease. The treatment effects of EDP1867 were most pronounced in the relapsing phase of disease indicating increased recovery after acute phase of disease. By the end of the study at day 42, EDP1867 treated mice had an overall lower cumulative EAE score compared to control (**Figure 4A**). Mice treated prophylactically with EDP1867 showed significantly reduced frequency of infiltrating inflammatory cells in the spinal cord compared to vehicle treated animals (**Figure 4B**). This suggests that prophylactic dosing of EDP1867 reduces the ability of immune cells to migrate and infiltrate into the CNS, resulting in improved motor functions and reduced disease severity.

Treatment in MS patients occurs after disease onset and treatments ultimately aim to control the severity of relapses. To test the ability of EDP1867 to control relapse/remission in the EAE mouse model we used a therapeutic dosing regimen for treatment two days after initial disease onset, which usually occurs around day 10 after immunization. Following analysis of relapse/remission, we observed significantly reduced cumulative disease scores at study termination compared to vehicle, and similar scores to the relevant human clinical dose of fingolimod (**Figure 4C**). At termination, we collected spinal samples from vehicle, EDP1867, and fingolimod treated animals and assessed for inflammatory foci and demyelination. While prophylactic dosing of EDP1867 reduced lymphocytic cell infiltration, therapeutic dosing did not show statistical differences between EDP1867- and vehicle-treated groups (**Supplementary Figures 4A–C**). However, following therapeutic dosing, EDP1867 significantly reduced demyelination of the spinal cord. Strikingly, EDP1867 significantly reduced the average demyelination score across the cervical, thoracic, and lumbar regions of the spinal cord (**Figure 4D**). Upon analysis of each section, we found that EDP1867 reduced demyelination in the thoracic and lumbar regions of the spinal cord, whereas the cervical region was not severely affected in this study (**Supplementary Figures 4D, E**). This suggests that although EDP1867 does not block the migration and infiltration of cells into the CNS when dosed therapeutically, the cells that are observed in the CNS have a lower capacity for inflammation-mediated demyelination and tissue damage.

We also performed RNA-Seq on the duodenum and colon from this study and found that EDP1867 elicited a robust transcriptional signal in the duodenum not seen in the colon. EDP1867 triggered gene expression changes in 1064 genes in the duodenum, which were substantially larger (more than 20-fold) than in the colon (46 genes) (**Supplementary Figure 5A**), indicating a site-dependent selectivity of the response. It is particularly intriguing that these immune-modifying gene expression responses to EDP1867 were restricted to the small intestine and not observed in the colon. Of the enriched cell types represented amongst the 590 genes elevated by EDP1867 in the duodenum, we observed an overrepresentation of intestinal epithelial cells (IECs) as well as a strong and statistically significant enrichment of immune cells across multiple lineages including myeloid cells, B cells and T cells (**Supplementary Figure 5B**). Consistent with this, we also found highly significant overrepresentation of immune-related pathways and processes especially those associated with negative regulation of T cell activation, B-cell receptor signaling, lymphocyte chemotaxis, cytokines and neutrophil-mediated immunity (**Supplementary Figure 5C**).

Collectively, these data demonstrate efficacy of EDP1867 in reducing pathology in two distinct IL-23/IL-17A axis driven inflammation models.

### EDP1867 Reduces Inflammation in FITC-Driven Contact Hypersensitivity

**Figure 5:**
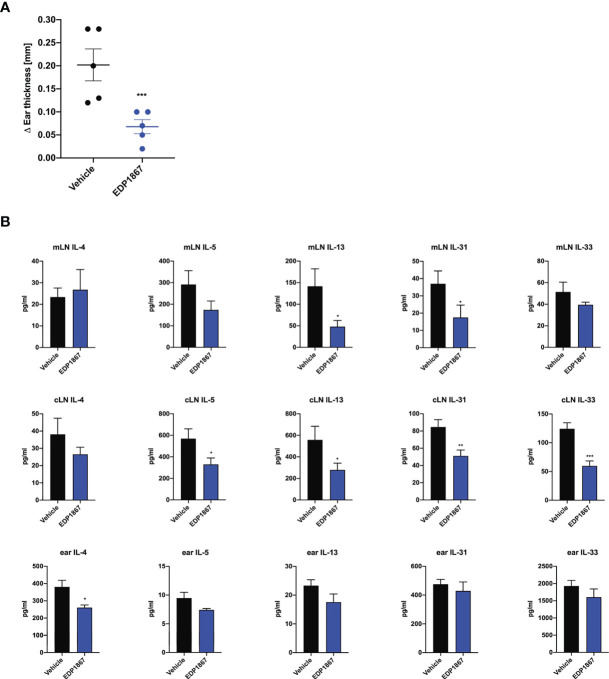
EDP1867 resolves inflammation in a Th2 cell driven atopic dermatitis model. FITC- driven AD. BALB/c mice were topically sensitized with 0.5% FITC, on day 1 and 2 and 6 days later challenged with 0.5% FITC on the ear. Mice were dosed daily orally with vehicle or EDP1867 (TCC – 2.16E+12). Ear inflammation was measured 24h post ear challenge on day 7. **(A)** Change in ear thickness. **(B)** At termination on day 7, total cells from mesenteric lymph nodes and cervical lymph nodes were restimulated with PMA for 48h. and ear tissue homogenates were prepared. Cytokines were measured from supernatants and homogenates by multiplex ELISA. Data are representative of 3 independent experiments (n = 5 mice/group). *p < 0.05, ***p < 0.0005, as determined by unpaired Student’s t-test.

Since we observed that EDP1867 is efficacious across different T cell driven disease models driven i.e. Th1 driven DTH and Th17 driven psoriasis and neuroinflammation, we postulated that EDP1867 has a broad inflammation resolving capacity. As atopic diseases have a strong Th2 component, we probed for EDP1867 efficacy in these diseases. Atopic dermatitis (AD) is a chronic inflammatory skin disease, driven by strong Th2 immune responses. It is a considered a primarily T cell-driven disease with a key role for cytokines IL-4, IL-5, IL-13, alarmin IL-33 (18, 19) and IL-31, a cytokine associated with itch (20) in AD pathogenesis. EDP1867 was tested in a murine model of contact hypersensitivity, using a hapten fluorescein isothiocyanate (FITC) to drive cutaneous inflammation (21, 22). In this model, mice are sensitized topically on back skin with FITC on day 1 and day 2 and receive an ear challenge on day 6 post sensitization. This model has similarities to human AD with a strong CD4^+^ T helper cell component and pathology associated with disease recapitulates features of acute AD lesions. FITC sensitization induced robust ear swelling. Oral treatment with EDP1867 significantly inhibited ear swelling compared to the vehicle-treated group (**Figure 5A**). We further investigated whether EDP1867 impacts production of cytokines driving inflammation. Ex vivo restimulation of cells from gut draining mesenteric lymph nodes (mLN) with PMA/Ionomycin showed decreased production of Th2 cytokines like IL-4, IL-5, IL-13, IL-31 and IL-33. Furthermore, *ex vivo* re-stimulation of cells from cervical lymph nodes (cLN), the draining lymph node for the site of inflammation, resulted in reduced production of IL-5 and IL-13, and homogenates made from ear tissue had reduced IL-4 in EDP1867 treated animals demonstrating the systemic effect of gut-restricted EDP1867 (**Figure 5B**). These findings confirm that EDP1867 has an immune resolving effect in inflammation by modulating levels of proinflammatory Th2 cytokines.

### EDP1867 Treatment Leads to Reduction of Local and Systemic Pro Inflammatory Cytokines *In Vivo*

**Figure 6:**
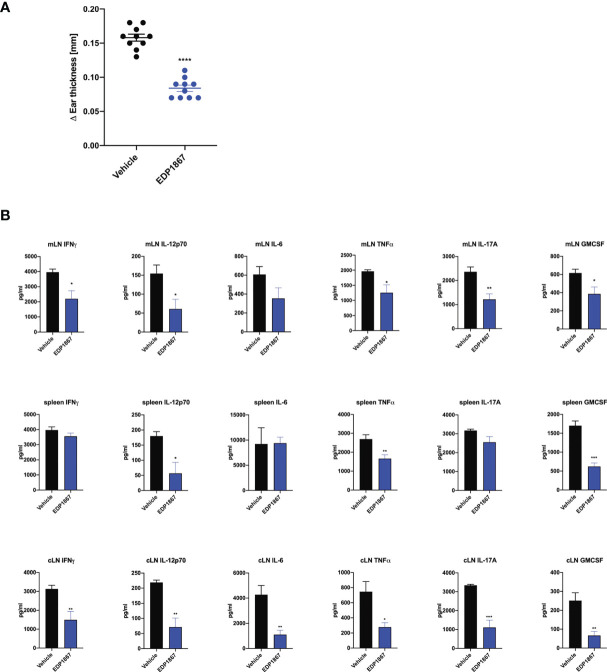
EDP1867 resolves inflammation in a delayed type hypersensitivity T cell driven disease model *in vivo*. KLH-DTH was induced as previously described. Mice were orally dosed daily from day 1 to 8 with a compressed tablet form of placebo or EDP1867. Ear inflammation was measured on day 9. **(A)** Change in ear thickness (n = 10 mice/group). **(B)** At termination on day 9 total cells from mesenteric lymph nodes and spleen were restimulated with PMA for 48h and total cells from ear draining lymph nodes were restimulated with KLH for 72h. Cytokines from supernatants were measured by multiplex ELISA. Data are representative from 2 experiments with n = 10/group. All data show mean ± SEM. *p < 0.05, **p < 0.001, ***p < 0.0005, ****p < 0.0001, as determined by unpaired Student’s t-test.

Given that EDP1867 exerts its effects locally in the gut to contain systemic inflammation, we sought to elucidate the impact of EDP1867 on downstream effectors such as cytokines and chemokines in the inflammatory milieu. A compressed tablet formulation of EDP1867, dosed daily for 9 days, was tested in the KLH DTH model as previously described. EDP1867 was efficacious in reducing ear inflammation in comparison to a group that received a placebo formulation (**Figure 6A**). *Ex vivo* analyses restimulation of cells from spleen and mLN taken from mice 24h post-ear challenge revealed significantly reduced production of proinflammatory cytokines including the Th1 cytokines IFNγ, TNFα, IL-12p70, IL-6, and the Th17 cytokines GMCSF and IL-17A. This suggested that treatment with EDP1867 had an anti-inflammatory effect on cells in the mLN as well as cells in periphery represented by splenic cells. (**Figure 6B**). *Ex vivo* restimulation with KLH to mimic antigenic stimulation of cells from cLN showed a similar trend of reduced pro-inflammatory cytokines TNFα, IL-6, IL-12p70, GMCSF, IL-17A and IFNγ in mice treated with EDP1867, compared to a control group treated with vehicle (**Figure 6B**).

### The Anti-Inflammatory Effects Driven by EDP1867 Require Trafficking of Immune Cells From the Gut to Periphery to Resolve Inflammation

To better understand the mechanism of action of the immunomodulatory effects of EDP1867 on the mucosal immune system, we employed a systematic approach to dissect the steps of the process. Firstly, we interrogated the interaction between EDP1867 and cells in the small intestine, including epithelial cells and dendritic cells. Several pattern recognition receptors are located on cell surfaces and of these, TLRs represent a major class (23). HEK-293 cells stably transfected with human TLR1/2/6, TLR2/6 and TLR1/6 and an NF-κB-inducible reporter gene were stimulated with different doses of EDP1867. EDP1867 was mostly detected by TLR2/6 with little to no detection by TLR1/6. To explore the role for TLR2 signaling *in vivo*, in the KLH DTH model as previously described, we observed that simultaneous dosing with EDP1867 and a TLR2 blocking antibody resulted in reduced efficacy in controlling ear inflammation in comparison with EDP1867 dosed with an isotype antibody (**Supplementary Figures 6A, B**). In C3HEJ mice that are genetically deficient for TLR4 (24), induced with KLH-DTH, treatment with EDP1867 was efficacious in lowering ear inflammation (**Supplementary Figure 6C**). This suggested recognition of EDP1867 was dependent on signaling *via* TLR2 receptor cascade and independent of TLR4 signaling.

Secondly, we investigated whether cells within the gut and periphery are traffic through the mLN. T cells traffic through mLN to encounter gut resident DCs that have sampled antigen as it passes through the lumen. Resident DCs could potentially interact with EDP1867 resulting in stimulation of *via* receptors including TLR2. The interaction between resident DCs and T cells within the mLN typically leads to activation of effector T cells which then enter systemic circulation, eventually migrating to peripheral tissues. Homing and retention of lymphocytes occurs through expression of specific adhesion molecules and chemokine receptors on lymphocytes in concert with spatial and temporal expression of specific ligands by stromal and structural cells in the mLN. LPAM-1 and CD62L are expressed on T and B cells and act as intestinal homing receptors to mediate migration of lymphocytes into mLN (25).

**Figure 7:**
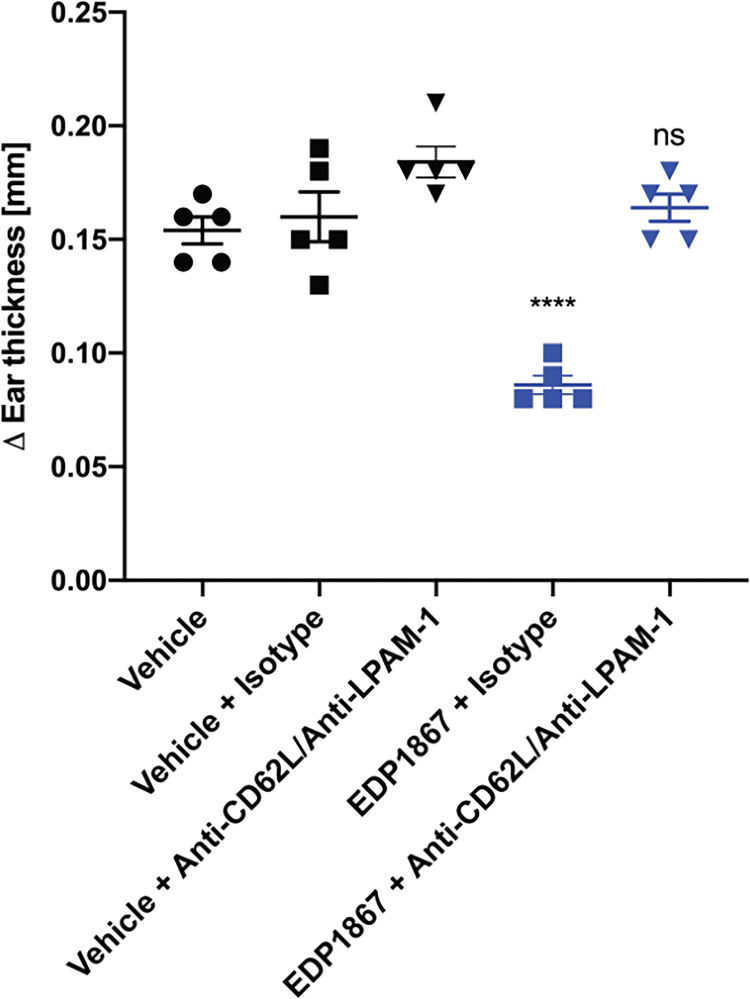
EDP1867 requires trafficking of gut immune cells to periphery to impact efficacy. Mice were treated with anti-LPAM1/CD62L blocking antibody or a rat IgG2a isotype antibody intraperitoneally on days 1,3, 5, and 7. Mice were challenged on day 14 and change in ear thickness was measured on day 15. All studies have n = 5 mice/group. Representative figure from n = 2 experiments. All data show mean ± SEM. **p < 0.01, ****p < 0.0001, ns, not significant as determined by ordinary One-Way ANOVA.

To test the importance of lymphocyte homing for the efficacy of EDP1867, we used an antibody combination that blocked binding of LPAM-1 and CD62L during the oral dosing period of EDP1867. We validated this antibody combination and showed that while the treatment significantly reduced the total cells within the mLN, after a one-week period to allow the antibodies to be cleared from the system, the DTH response itself was not inhibited. After antibody treatment, we found a reduced frequency of total CD45+ cells, but specifically, we observed a significant loss of CD3^+^, CD3^+^CD4^+,^ and CD3^+^CD8^+^ lymphocytes, as well as a reduction in B cells. Conversely, the number of CD11b+ cells within the mLN was significantly increased, mostly likely to compensate for the loss of lymphocytes within this tissue (**Supplementary Figures 7A–E**). Simultaneous blockade of both receptors only during the period of oral dosing with EDP1867 resulted in loss of efficacy with EDP1867 treatment in comparison with a control group that received EDP1867 with an isotype antibody (**Figure 7**). These results demonstrate that efficacy of EDP1867 was contingent upon the presence of lymphocytes in lymph nodes during the period of transit of EDP1867 through the gastrointestinal tract. This suggests that while administration of anti-LPAM-1 and anti-CD62L can block lymphocyte migration to all peripheral lymph nodes, the lymphocytes specifically within the gut-draining mLN are likely to be important for mediating the anti-inflammatory effect of EDP1867.

Taken together, these data demonstrate that EDP1867 has a direct interaction with sentinel cells within the gut, resulting in modulation of lymphocytes, specifically within the mLN, that facilitates a modification of these cells towards a function of inflammation resolution.

### Adoptive Transfer of CD4+ T Cells From EDP1867 Treated Donors Leads to Inflammation Resolution *In Vivo*

**Figure 8:**
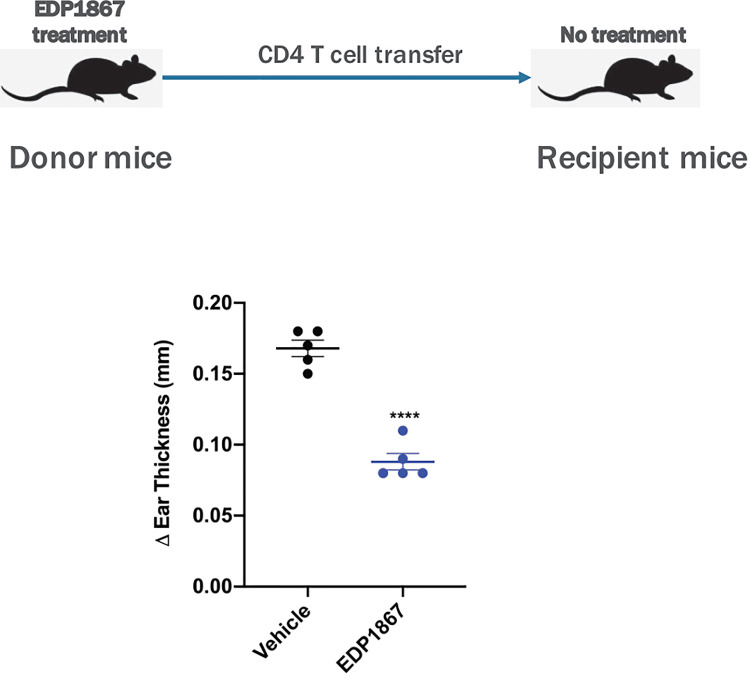
Adoptively transferred EDP1867-treated CD4 T cells mediate efficacy in DTH. Adoptive transfer of CD4+ T cells from mice treated with EDP1867. Mice with DTH were treated with EDP1867 for 4 days and then CD4 T cells from these treated animals were adoptively transferred into recipient immunized animals that were not dosed with EDP1867. Representative figure from n = 2 experiments with 5mice/group in each experiment; ****p < 0.0001, as determined by Ordinary one-way ANOVA.

Based on our data confirming a role for EDP1867 in inflammation resolution, we further investigated the specific role for CD4+ T cells in this context. Using an adoptive cell transfer model, CD4^+^ T cells from EDP1867 treated KLH-immunized donor mice were transferred into KLH-immunized recipient mice that were not dosed with EDP1867. 4 days post adoptive transfer, recipient mice were challenged with KLH intradermally in the ear to elicit local inflammation. Recipient mice that received CD4^+^ T cells from EDP1867 treated donors had significantly reduced ear inflammation in comparison with recipients that received CD4^+^T cells from vehicle treated mice (**Figure 8**). These data suggest that treatment with EDP1867 might confer anti-inflammatory functions to the CD4^+^ T cells that are subsequently transferred and these cells are sufficient to drive an anti-inflammatory response in the recipient mice.

## Discussion

In this report we describe the development of EDP1867, an orally administered, gut-restricted strain of *Veillonella parvula* for the treatment of inflammatory diseases. Animal models and human diseases tend to be arbitrarily defined in pathway specific terms Th1, Th2, Th17, innate or adaptive. However, induction of inflammation is not generally pathway specific. While existing therapeutic interventions do indicate some pathway biases in disease pathogenesis, such as TNF inhibitors in rheumatoid arthritis or IL17 inhibitors in psoriasis, our data demonstrate that orally administered EDP1867 is effective in murine models of Th1, Th2 and Th17 inflammation. This shows that the functional connections radiating from the small intestinal mucosa can induce systemic resolution of broad inflammatory mechanisms leading to restoration of immune homeostasis.

EDP1867 is efficacious in *in vivo* models of skin inflammation and central nervous system inflammation. Given the more acute nature of the skin challenge models, we wanted to test the ability of EDP1867 to inhibit inflammation in a more chronic disease model, such as EAE. It has been previously reported that a commensal strain of *Prevotella histicola* was able to inhibit inflammation in a human leukocyte antigen (HLA) class II transgenic mouse model (7). This inhibition of inflammation correlated with an increase in Tregs, reduction in blood-brain barrier (BBB) permeability, and a decrease in CNS-infiltrating inflammatory T cells. In our EAE study, we observed that although therapeutic treatment with EDP1867 led to a reduction in disease score, as well as in demyelination, CNS-infiltrating cell numbers were not changed. Although we did not determine the nature of the infiltrating cells, the reduction in inflammation suggests that the pro-inflammatory nature of the infiltrating cells was altered. It is therefore possible that treatment with EDP1867 did not reduce BBB permeability or prevent migration of cells to the CNS, but was able to reduce the inflammatory nature of the infiltrating cells themselves.

EDP1867 exerts its anti-inflammatory effects after being rendered non-viable by gamma-irradiation. Because EDP1867 is non-viable, colonization of the intestine by EDP1867 is not required for its pharmacological activity. We observed rapid transit through the gastrointestinal tract within hours of administration and treatment with EDP1867 caused no changes in the microbiome. The suggests that the mechanism of action of EDP1867 is entirely dependent on direct interactions with host intestinal cells, in part mediated through TLR-2. This is distinct from reports of live bacterial therapeutics altering the ecology of colonic microbiota (4). While a number of previous studies use a reductionist approach to identify immune effects of bacterial species in germ free animals (26), all our experiments were done in specific pathogen-free animals with intact intestinal microbiomes to represent the complex relationships in the gut- microbial environment. The dose-dependent effects of EDP1867 were superimposed on this microbial background.

The dependence on IL-10 for the efficacy of EDP1867 in the delayed-type hypersensitivity model is a clue to the differences between the effects mediated by the mucosal immune system and established therapeutics based on inhibition of pro-inflammatory mediators. Systemic administration of IL-10 as an anti-inflammatory therapy has not been successful to date, despite the long-standing awareness that it is a critical mediator of the resolution of inflammation. The pharmacological demonstration of the role that IL-10 plays in the activity of EDP1867 shows an alternative way to harness its anti-inflammatory effects. It further bolsters the fact that local gut effects in response to specific immunomodulatory bacterial strains can regulate immune responses at distal sites in concordance with previous reports (7).

Additionally, EDP1867 is sensed in the gut and can trigger robust gene expression alterations in the small intestine that characterize early insights into immune sensing, migration and response modifying programs across a broad subset of B-, T- and myeloid lineages. Enriched pathways and processes elicited by EDP1867 impacted immune modulation, regulation of T-, B- and myeloid cells and chemotaxis, which represent alterations in immune responses occurring locally in the gut that can potentiate systemic shifts towards immune homeostasis more distally. Interestingly, this local response appears to be restricted to the small intestine, which is informative that key regulators and potentially, drivers of systemic effectors may originate in the small intestine.

There are two main conclusions from this work. The first is that the intestinal mucosa can act as a central controller of systemic inflammation, operating at a distance in response to signals generated by substances passing through the gut. The effects appear to represent broad-based resolution of inflammation that re-establishes normal homeostatic inflammatory status across multiple pathways.

The second is that a single strain of non-viable bacteria that does not colonize the gut can mediate this peripheral anti-inflammatory effect, which suggests a therapeutic approach for common inflammatory diseases. Resolution of inflammation by a non-absorbed oral agent acting *via* the mucosal immune system has the potential to create a new class of safe and effective medicines that can be manufactured at reasonable cost for the treatment of inflammatory diseases suffered by millions of patients. These results support a series of clinical studies currently ongoing with EDP1867.

## Materials And Methods

### Mice

Female BALB/c and C57BL/6 mice (6-8 weeks old) were purchased from Taconic Farms. Animals were housed in specific pathogen–free conditions in a vivarium (5 mice per cage), and all experiments were performed under Institutional Animal Care and Use Committee (IACUC) approved protocols and guidelines at Avastus Preclinical Services (Cambridge, MA). Female SJL mice (8-10 weeks old) were purchased from Jackson Labs for EAE experiments which were performed under IACUC approved protocols at Hooke Laboratories (Lawrence, MA). Mice were allowed to acclimate in the vivarium for 1-2 weeks prior to the start of experiments. PicoLab Rodent Diet 20 was provided and autoclaved water *via* sipper bottle, given *ad libitum* and checked daily.

### Strains of Microorganisms

Three individual strains of *Veillonella* species were obtained for this study. All strains were purified *via* single colony isolation method and maintained in Evelo Biosciences Inc. Bacterial Library. Strain’s identity was confirmed by 16S rDNA sequence comparisons and by the whole genome sequencing. *Veillonella parvula* (EDP1867) and *Veillonella tobetsuensis* were isolated from a fresh ileostomy sample and a pre-colonoscopy sample of two IBD patients in remission. *Veillonella atypica* was isolated from a fresh subgingival sample of a healthy volunteer. The study protocols EVB-006-01 and EVB006-03 “Study of the human microbiome in volunteers” and EVB-006-02 “Study of the oral human microbiome in volunteers” were approved by the IntegReview IRB, 3815 S. Capital of Texas Hwy, Suite 320, Austin, TX 78704; informed consents were obtained from the volunteers.

### Microbial Biomass Growth Conditions

All strains were grown in in-house developed semi-defined Soy Peptone-Yeast Extract medium supplemented with Na-L-Lactate as a major carbon source, mineral elements, and L-cysteine-HCl as a reducing agent. All microbial cultures were incubated under anaerobic conditions at 37°C for 16-24 hours before harvesting. Bacterial biomass was concentrated by centrifugation at 7000g for 20 min at 10°C, resuspended in anaerobic glycerol or yeast extract-sucrose solutions and distributed into 1.8ml cryovials at 1.2-1.5ml volume under anaerobic conditions. Cryovials were immediately frozen in liquid N2 and stored at -80°C.

EDP1867 was prepared in different forms- powders and frozen biomasses and was characterized by TCC method. Bacterial total cells count (TCC) was enumerated by Coulter Counter Multisizer4e. In powders TCC varied from 4.0e+11 to 2.2e+12 cells/g. In biomasses TCC varied from 2.8e+10 to 8.5e+10 cells/ml. Bacterial biomass identity was confirmed by 16S rDNA sequencing. Powder was produced following in-house developed fermentation and lyophilization protocols and stored in sealed mylar bags inside desiccator at 4°C.

EDP1867 aliquots were subjected to 25 kGy Gamma Irradiation treatment at Sterigenics U.S., LLC. EDP1867 aliquots were characterized by TCC and VCC methods before and after Gamma Irradiation. Total cell number did not change, while there were no viable cells left after the treatment.

### Dosing With EDP1867 and Controls *In Vivo*

For each *in vivo* study, EDP1867 aliquots were distributed into plastic test tubes with caps and stored at 4°C. Mice were treated orally with EDP1867 (4.0E+11 to 2.2E+12 cells/g PO- specific TCC is noted in figure legends) or vehicle control (anaerobic sucrose, PO) for duration of different models as described in figure legends. Dexamethasone (1mg/kg, i.p., Sigma) was used as a positive control unless otherwise specified. For EAE studies, fingolimod (1 mg/kg, PO, Tocris Biosciences) was dosed daily.

For blockade of IL-10R, mice were injected IP with 100 uL of either anti-IL-10R (Bio X Cell; Clone 1B1.3A; Cat# BE0050) or Rat IgG1 isotope control (Bio X Cell; Clone HRPN; Cat# BE0088) at 2 mg/mL. Mice were treated on days 0, 3, and 6.

For blockade of TLR2, mice were injected IP with 100 uL of either anti-TLR2 (*In vivo*gen; Clone T2A; Cat# mab2-mtlr2) or Mouse IgG1 isotope control (Bio X Cell; Clone MOPC-21; Cat# BE0083) at 2 mg/mL. Mice were treated days 0, 3, and 6.

For blockade of lymphocyte migration, the KLH DTH study was performed as described below with the following alterations. Mice were treated with either Rat IgG2a isotype control (100 μL; 5μg/mL; Clone 2A3; Bio X Cell) or anti-LPAM-1 and anti-CD62L (100 μL; 2.5 μg/mL each; Clones DATK32 and Mel-14 respectively; Bio X Cell) by intraperitoneal injections on days 1, 3, 5, and 7. Mice were dosed with EDP1867 on days 5-8. After a one week washout period, baseline ear measurements were taken on Day 14, ears were challenged with KLH intradermally, and ears were measured 24 hours later on day 15.

### Delayed-Type Hypersensitivity Mouse Model

Mice were immunized with 50 μl of emulsion of keyhole limpet hemocyanin (KLH) in Complete Freund’s Adjuvant (CFA) on four sites on the back. 8 days later, recipient mice were challenged with KLH (10 μg/10 μl) intradermally in the ear. Ear measurements were recorded 24 hours post ear challenge using digital calipers. Change in ear thickness was expressed as ear thickness at day 7 minus ear thickness at baseline.

For the adoptive transfer DTH, cells were isolated from spleens and all lymph nodes of C57BL/6 mice and single cell resuspensions were made. CD4^+^ T cells were isolated using EasySep Mouse CD4+ isolation kits (StemCell Technologies, Cat#19852). 1.5-2X10^7^ cells resuspended in 200 μl of PBS were injected into recipient mice. 4 days after adoptive transfer of cells, recipient mice were challenged with KLH (20 μg/20 μl) intradermally in the ear. Ear measurements were recorded 24 hours post ear challenge.

### Imiquimod-Induced Psoriasis-Like Skin Inflammation Protocol

Mice were sensitized topically with 20 mg imiquimod cream (Aldara; 3M Pharmaceuticals, St Paul, MN, USA) on ears daily for 7 consecutive days. Ear measurements were taken daily using digital calipers and scores were reported as change in ear thickness calculated as ear score daily minus baseline ear score on day 1 prior to sensitization with imiquimod.

### Experimental Autoimmune Encephalomyelitis

Female SJL mice (8-10 weeks old) were subcutaneously injected at four sites with myelin proteolipid protein (PLP)_139-151_ in CFA emulsion (0.05 mL/injection site; ~0.5mg PLP PLP_139-151_/mL; Hooke Laboratories; EK-2120). Following immunization, EAE induction was completed by intraperitoneal injections of pertussis toxin (6 μg/mL; 0.1 mL/mouse) within 2 hours of immunization. Mice were randomized into groups and monitored for EAE clinical score over the course of 42 days. Disease progression was scored blinded of treatment or prior measurements. Disease severity was scored using standard EAE criteria: 0 (normal); 1 (loss of tail tone); 2 (hind limb weakness); 3 (hind limb paralysis); 4 (hind limb paralysis and forelimb paralysis or weakness); 5 (morbidity/death). Mice were observed daily for clinical symptoms. Mice were euthanized if they had a score of 4 for 2 days, and a score of 5 was recorded for remainder of the study for these animals.

### Terminal Tissue Collection and Histology

After euthanasia at the end of the study, EAE mice were perfused with 5–10 mL PBS and the spinal column was extracted from the base of the skull to the beginning of the pelvic bone. Spinal columns were then drop-fixed in 10% neutral buffered formalin and stored horizontally for 48 hours. After fixation, spinal columns were treated in mild formic acid decalcification solution (Immunocal-Statlab, Fisher Scientific, #141432) overnight (12–24 hours) at room temperature. Spinal columns were then trimmed into 4 mm-thick cervical, thoracic, and lumbar segments and processed using a Sakura Tissue Tek VIP 5 by graded alcohol dehydration, cleared in xylene, and finally infiltrated with paraffin. After processing, spinal column segments were embedded into paraffin blocks. Paraffin blocks were then sectioned at 4 μm on charged slides, air-dried overnight and stained with Hematoxylin and Eosin according to standard automated H&E protocol (Tissue-Tek Prisma) and then cover slipped (Tissue-Tek Glass). Prepared tissue sections were then imaged using a NanoZoomer 2.0 HT (Hamamatsu) at 20X magnification.

Slides were also stained with Anti-MBP immunohistochemistry performed using the Ventana Discovery XT (Roche/Ventana, Tucson AZ) automated Platform. Slides were deparaffinized and rehydrated. Endogenous Biotin blocking was performed online 4 min followed by 32 min incubation in the anti-MBP primary (Abcam. Ab40390 (1ug/ml) LotGR3264120-1) at a 1:1000 dilution. The biotinylated GtxRb secondary (Invitrogen 65-6140 LotTD268284) was incubated for 32 minutes at a 1:1000 dilution. HRP based detection was performed online using the standard DABMAP Kit (Roche/Ventana 760-124) followed by a Hematoxylin counterstain. Slides were then dehydrated through graded alcohols cleared in Xylene and cover slipped. Stained Slides were scanned on Nanozoomer 2.0 HT (Hamamatsu) at 20x magnification and images were analyzed by a pathologist.

### Histological Analysis of Spines

For each spine, one anti-MBP-stained slide and one H&E-stained slide was prepared and analyzed. Each slide contained a section with samples from lumbar, thoracic, and cervical spinal cord (3 samples). Histological analysis was performed blind to groups and included count of inflammatory foci, count of apoptotic cells, and demyelination score. All analysis was performed by a pathologist blinded to the experimental groups and all clinical readouts.

*Count of inflammatory foci – spinal cord* -Inflammatory foci of approximately 20 cells were counted in each H&E-stained section. When inflammatory infiltrates consisted of more than 20 cells, an estimate was made of how many foci of 20 cells were present.

*Estimation of demyelinated area – spinal cord* - The demyelination score represents an estimate of demyelinated area for each section as follows:

0 – no demyelination (less than 5% demyelinated area)
1 – 5 to 20% demyelinated area
2 – 20 to 40% demyelinated area
3 – 40 to 60% demyelinated area
4 – 60 to 80% demyelinated area
5 – 80 to 100% demyelinated area

For anti-MBP-stained slides, the size of the demyelinated area was estimated based on less intense brown staining of myelin.

### FITC-Induced Allergic Inflammation

Backs of female BALB/c mice were shaved and on days 1 and 2 400μl of 0.5% FITC solution (dissolved in acetone: dibutyl phthalate, 1:1, v/v) was painted on the shaved skin. On day 6, baseline ear measurements were taken and then mice were challenged with 20 μl 0.5% FITC on the right ear. On day 7, ear thickness was measured 24 hours post FITC challenge using digital calipers (Fowler). Change in ear thickness was expressed as ear thickness at day 7 minus ear thickness at baseline.

### *Ex Vivo* Re-Stimulation Assays

Ear-draining cervical lymph nodes (CLNs), gut draining mesenteric lymph nodes (MLNs) and spleens were harvested at terminal time points from various studies and collected into 0.5 ml of cold, complete-RPMI (10% FBS, 1x Glutamax, 1 mM sodium pyruvate, 100 mM HEPES, 1x non-essential amino acids, 1x beta-mercaptoethanol, 1x antibiotic-antimycotic) (all reagents from Gibco). Single cell suspensions were prepared (spleens were RBC lysed with ACK lysing buffer) and 200,000 cells/well were plated. Cells were stimulated *ex vivo* with either LPS (200 ng/ml, *In vivo*gen) or PMA (eBioscience) for 48 hours, or KLH (50 μg/ml, Sigma) or OVA (50 μg/ml) for 72 hours at 37°C and 5% CO2. Supernatants were collected at the end of stimulations and used for multiplex ELISAs of cytokine levels using Meso Scale Discovery kits. Ear tissues were dissociated in 250μl T-PER buffer (Thermo Scientific) containing Halt Protease (Thermo Scientific) and protein was quantified with BCA kit (Thermo Scientific). 100μg of protein was used to measure cytokine levels using MSD kits.

#### Quantitative PCR

Total RNA was extracted from skin tissue samples at terminal time point on day 8 using the RNEasy Mini kit (Qiagen). Using 100 ng of total RNA, qPCR was run using the One step RNA-to-cT (Applied Biosystems*).* Taqman primers for *Il17A (*Mm00439618_m1)*, Il17f (*Mm00521423_m1)*, Defb3 (*Mm04214158_s1), and *Gapdh(*Mm99999915_g1) were ordered from ThermoFisher Scientific. *Il17A, Il17f, Defb3*, and *Gapdh* mRNA levels were measured by real-time quantitative PCR analysis using the ABI PRISM 7700 sequence detection system (Applied Biosystems). Cytokine and GAPDH fold change in comparison to a naïve control were calculated by delta delta Ct method.

### EDP1867 Fluorescent Labeling Fluorescent Labeling

Samples were always kept in ice unless specified. To remove excipients, 200 mg of EDP1867 drug substance (powder formulation) were resuspended and extensively washed by repeated centrifugation/resuspension (4 min, 9000 x g, 4°C) using sterile labeling buffer (2x PBS buffer pH 8.3). Final microbial cell pellets where resuspended in 1 mL final volume using labeling buffer. The fluorescent labeling reaction was started by addition of IRDye800-NHS Ester (LICOR Biosciences) at 50 µM final concentration (from 10 mM dye stock dissolved in DMSO). The reaction was allowed to continue for 1.5 hours at 22°C with gentle agitation, followed by overnight incubation at 4°C. Non-reacted dye was removed by repeated steps of centrifugation/resuspension in sterile PBS buffer until no fluorescence was detected in the supernatant. Fluorescently labeled microbial cells were resuspended in sterile PBS buffer and cell concentration was quantified using coulter counter instrument (Beckman). The molar concentration of IRDye800^®^ covalently attached to microbial cells was quantified by UV-Vis absorbance and using the dye’s molar extinction coefficient 240.000 M^-1^cm^-1^ at 778 nm in PBS buffer (LICOR Biosciences). A suspension of unlabeled bacterial cells was used to subtract any light scatter contributions.

### Biodistribution Studies

One week prior to experiments, Balb/C female mice were place under a specific low fluorescence diet without chlorophyl (AIN-93G, Bio-Serv). Mice were randomized in groups of 3 animals and orally dosed with 100 µl PBS buffer containing 1 x 10^9^ total EDP1867 cells covalently labeled with IRDye800, as well as an equimolar dye amount of IRDye680RD-Carboxylate (LICOR Biosciences) as a free-dye control. At each time point within each experimental replicate, mice were sacrificed using CO_2_ and cervical dislocation followed by careful removal of the complete Gastrointestinal Tract (GIT), mesenteric lymph nodes (MLN), liver, spleen, heart, and lungs. These were imaged in a tray using a whole-animal fluorescence imaging instrument (Pearl^®^, LI-COR Biosciences). Tissues were imaged using the 800 nm emission channel (EDP1867), the 700 nm emission channel (free dye control), and the standard white epi-illumination channel (overall tissues). After imaging, the individual organ weights were recorded. Fluorescence images were used to quantify the distribution of EDP1867 signal in the GIT as well as the different organs. Tissues were manually outlined, and the fluorescence signals quantified using image analysis software (Image Studio^®^, LICOR Biosciences). The fluorescence signal from different organs was divided by the recorded tissue weight in order to normalize the data. Data points corresponded to mean ± standard deviation values.

### *In Vitro* Assays

For *in vitro* assays thawed bacterial biomass was serially diluted in RPMI degassed medium inside anaerobic chamber (Coy Lab Products, USA) to reach approximately 2E+6 bacterial cells/ml. 100000 bacterial cells were added to 200000 purified human immune cells per each 96-well manually or by using automated Liquid handler Biomek 4000 (Beckman Coulter) inside custom-built Coy Anaerobic chamber (Coy Lab Products, USA). The co-cultures were incubated for 24 hrs under micro-oxic conditions (1% O2, 5% CO2, balanced by N2). After incubation cell supernatants were collected and Luminex or MSD technology was used to measure pro-and anti-inflammatory cytokine production.

### Human PBMC Assay

Freshly isolated PBMCs from 6 different human donors were cultured at 100,000 PBMCs/well in 100μl and incubated at 37°C overnight. The following day 75ul of supernatant was removed and replaced with fresh antibiotic free media. Microbes were added in anaerobic conditions and flushed with 1% oxygen. Plates were incubated for 24hrs in an anaerobic box at 37°C +5% CO2. After 24 hours, plated were centrifuged and supernatants collected to assay cytokine levels using MSD assays. 87 unique bacterial strains representative of human microbiome strains that were isolated from numerous human fecal and oral samples, or probiotic mixtures. The bacterial screening panel included representative species from 17 taxonomic families and 25 genera that belong to Actinobacteria, Firmicutes, Proteobacteria, and Bacteroidetes Phyla. The tested species represented the following taxonomic families: *Akkermansiaceae, Bifidobacteriaceae, Clostridiaceae, Enterobacteriaceae, Enterococcaceae, Erysipelotrichaceae, Eubacteriaceae, Lachnospiraceae, Lactobacillaceae, Oscillospiraceae, Paenibacillaceae, Peptostreptococcaceae, Porphyromonadaceae, Prevotellaceae, Rikenellaceae, Streptococcaceae, Veillonellaceae. *These families were represented by *Akkermansia, Alistipes, Anaerostipes, Bifidobacterium, Blautia, Butyricicoccus, Clostridium, Enterococcus, Erysipelatoclostridium, Escherichia, Eubacterium, Faecalimonas, Hungatella, Lachnoclostridium, Lactobacillus, Lactococcus, Mediterraneibacter, Paenibacillus, Parabacteroides, Prevotella, Roseburia, Ruminococcus, Streptococcus, Turicibacter, Veillonella *genera.

### Human Macrophage Assay

Freshly isolated PBMCs from 6 different human donors were used for isolation of CD11b+ macrophages. PBMCs were washed in 10ml MACS buffer, spun down and resuspended at a concentration of 10^7^ total cells per 80μl. Anti-CD11b+ beads were added (20μl per 10^7^ cells) and the cell suspension was incubated at 4°C for 15 minutes. Following incubation, cells were washed and resuspended in MACS buffer and CD11b+ cells were isolated using magnetic separation as per manufacturer’s protocol (Miltenyi). Isolated cells were further cultured at 100,000 CD11b+ cells/well in 100μl and incubated at 37°C overnight. The following day, contents in the well were mixed to bring non-adherent cells into suspension and 75ul of supernatant was removed and replaced with fresh antibiotic free media. Microbes were added in anaerobic conditions and flushed with 1% oxygen. Plates were incubated for 24hrs in an anaerobic box at 37°C +5% CO2. After 24 hours, plates were centrifuged, and supernatants collected to assay cytokine levels using MSD assays.

### Human Dendritic Assay

Freshly isolated PBMCs from 6 different human donors were used for isolation of blood dendritic cells. PBMCs were washed in 10ml MACS buffer, spun down and resuspended at a concentration of 10^8^ total cells per 300μl. FcR blocking reagent and non-DC Depletion cocktail were added (100μl per 10^8^ cells) and the cell suspension was incubated at 4°C for 15 minutes. Following incubation, cells were washed and resuspended in MACS buffer and run through the magnetic LD column for a depletion separation. The non-DC cells were retained in the magnetic column whereas the unlabeled cells (DCs) were collected as flow through. The unlabeled cells (DCs) were washed and labeled with a DC enrichment cocktail (100uL per 10^8^ cells) and the cell suspension was incubated at 4°C for 15 minutes. Following incubation, cells were washed and resuspended in MACS buffer and run through a magnetic MS column for a positive selection. Positively Isolated cells were further cultured at 30,000 Dendritic cells/well in 100μl and incubated at 37°C overnight. The following day, 75ul of supernatant was removed and replaced with fresh antibiotic free media. Microbes were added in anaerobic conditions and flushed with 1% oxygen. Plates were incubated for 24hrs in an anaerobic box at 37°C +5% CO2. After 24 hours, plates were centrifuged, and supernatants collected to assay cytokine levels using MSD assays.

### Statistical Analysis

The data were expressed as mean ± standard deviation. Statistical significance between groups was compared using the one-way ANOVA compared with sucrose-treated control. For statistical analyses of EAE data, the following tests were used for each readout: EAE incidence, Chi-square test; Mean day of EAE onset, 2-tailed Student’s t-test; Median day of EAE onset, Wilcoxon’s survival test; Average clinical score, 2-tailed Student’s t-test; Average end clinical score, Wilcoxon’s non-parametric test; Mean maximum score (MMS), Wilcoxon’s non-parametric test; Average weight gain/loss, 2-tailed Student’s t-test; End weight gain/loss, 2-tailed Student’s t-test; Incidence of EAE relapse, Chi-square test; MMS of relapses, Wilcoxon’s non-parametric test; MMS of relapse period, Wilcoxon’s non-parametric test. Significance was assigned at p < 0.05, All statistical tests were performed using Prism 8 (GraphPad Software, San Diego, CA, USA).

## Data Availability Statement

The raw data supporting the conclusions of this article will be made available by the authors, without undue reservation.

## Ethics Statement

The animal study was reviewed and approved by Avastus Institutional Animal Care and Use Committee; Avastus Preclinical Services, 44 Spinelli Place, Cambridge, MA 02138.

## Author Contributions

KR, AC, KB, SA, AN, HP, MS, CJHD, TG, and AI conceptualized and designed studies. KR, TC, KB, MA, IW, AA, PPa, SA, CJHD, FBR, AN, WC and DR performed experiments and analyzed data. VK, TR, EK, PPr generated material to be tested in experiments KR, AI, and MB wrote the manuscript. All authors contributed to the article and approved the submitted version.

## Conflict of Interest

All authors are employees and shareholders of Evelo Biosciences, which is sponsoring the clinical development of EDP1867.

## Publisher’s Note

All claims expressed in this article are solely those of the authors and do not necessarily represent those of their affiliated organizations, or those of the publisher, the editors and the reviewers. Any product that may be evaluated in this article, or claim that may be made by its manufacturer, is not guaranteed or endorsed by the publisher.
