# Supplementary material for: Regulation of Peripheral Inflammation by a Non-Viable, Non-Colonizing Strain of Commensal Bacteria
Source: Front Immunol. 2022 Feb 2;13:768076. doi: 10.3389/fimmu.2022.768076 (PMC8847375; doi:10.3389/fimmu.2022.768076)
Supplement: Supplementary file 1 [file DataSheet_1.pdf]

EVELO

1867 manuscript – Supplementary Figures

List of supplementary figures

Supplementary Fig. 1 – In vitro 87 unique bacterial strains screen IP10, TNFa and In vitro assays *Veillonella* screen in human primary cells

Supplementary Fig. 2 – No detection of 1867 in various organs -Biodistribution

Supplementary Fig. 3 – No changes to microbiome

Supplementary Fig. 4 –no change in inflammatory loci with therapeutic treatment with EDP1867 in EAE

Supplementary Fig. 5 – RNA Seq of duodenum and colon samples from mice with therapeutic treatment with EDP1867 in EAE

Supplementary Fig. 6 –TLR2 dependent and TLR4 independent

Supplementary Fig. 7-- Validating of loss of migrating cell upon inhibition with combination of anti-LPAM-1 and anti--CD62L

Supplementary Figure 1. EDP1867 induces comparable levels of IL-10 *in vitro* when stimulated with different strains of *Veillonella* in human primary cell assays

S1A

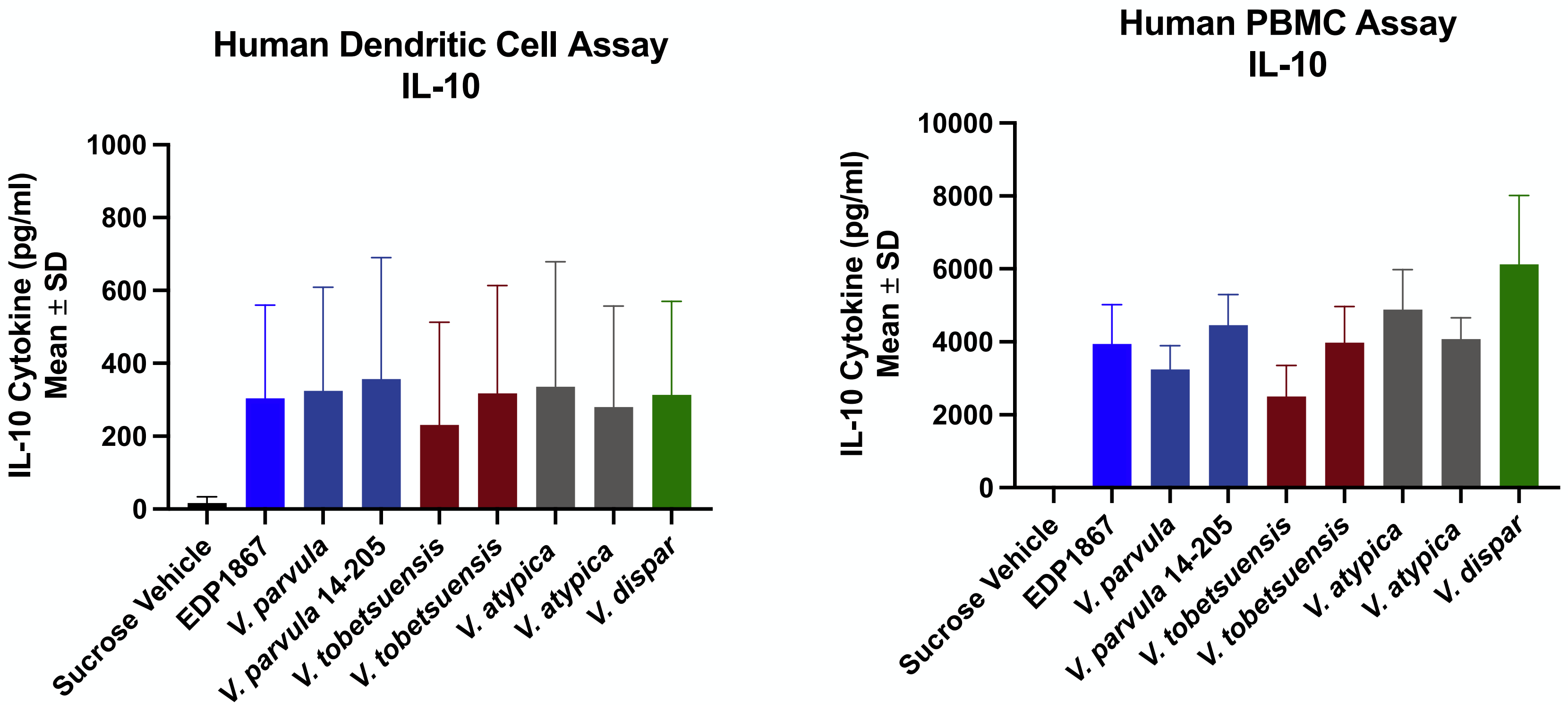

Human Dendritic cells and PBMCs were stimulated with different gamma irradiated *Veillonella* strains for 24h and flushed with 1% oxygen and supernatants were collected to test for IL-10 by MSD. Data represented as mean+SEM of collective data from 3 independent human donors.

EVELO

Supplementary Figure 1. EDP1867 is one of the highest inducers of IL-10 *in vitro* when stimulated with different strains of *Veillonella* in an inflamed human antigen presenting cell assay

S1B

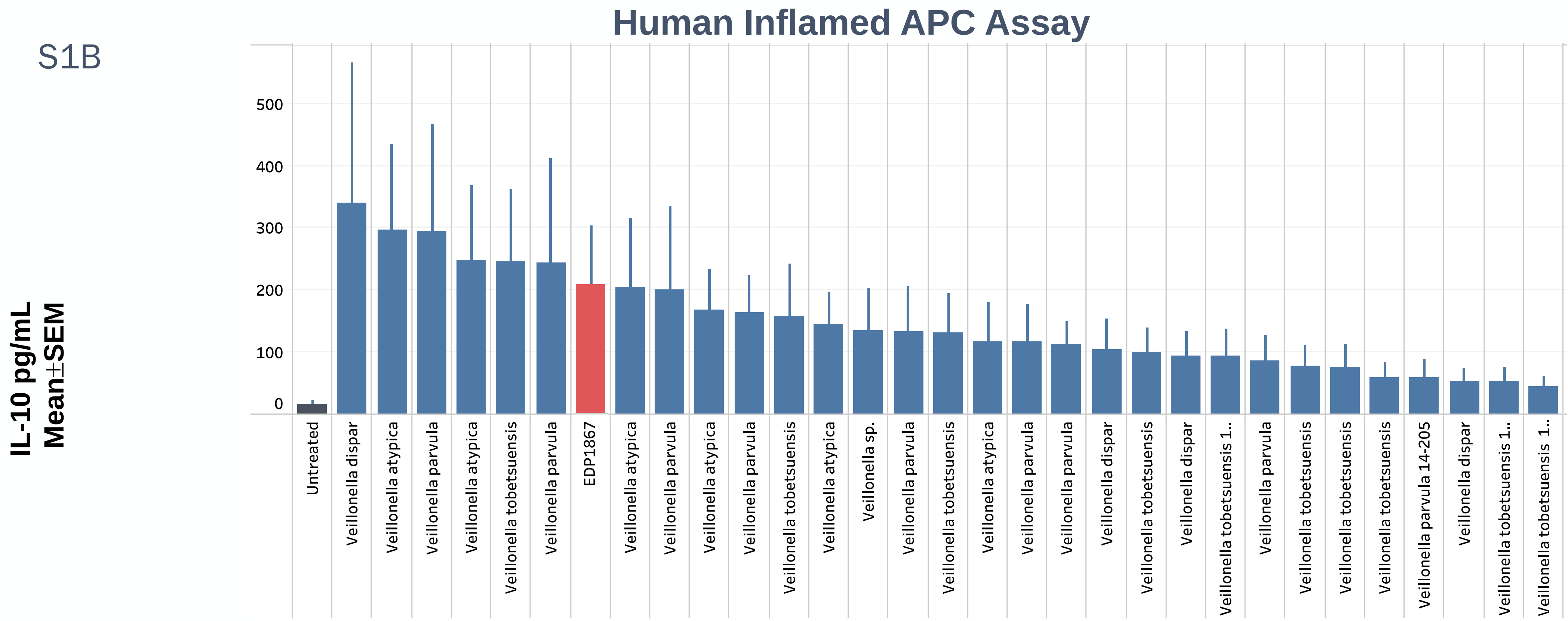

Human inflamed APCs were stimulated with different gamma irradiated *Veillonella* strains for 24h and flushed with 1% oxygen and supernatants were collected to test for IL--10 by Luminex. Data represented as mean+SEM of collective data from 6 independent human donors.

EVELO

Supplementary Figure 1. EDP1867 reduced IP-10 and TNFa when stimulated with strains of gamma irradiated *anaerobic bacteria* in *in vitro* human primary cell assays

S1C

IP-10

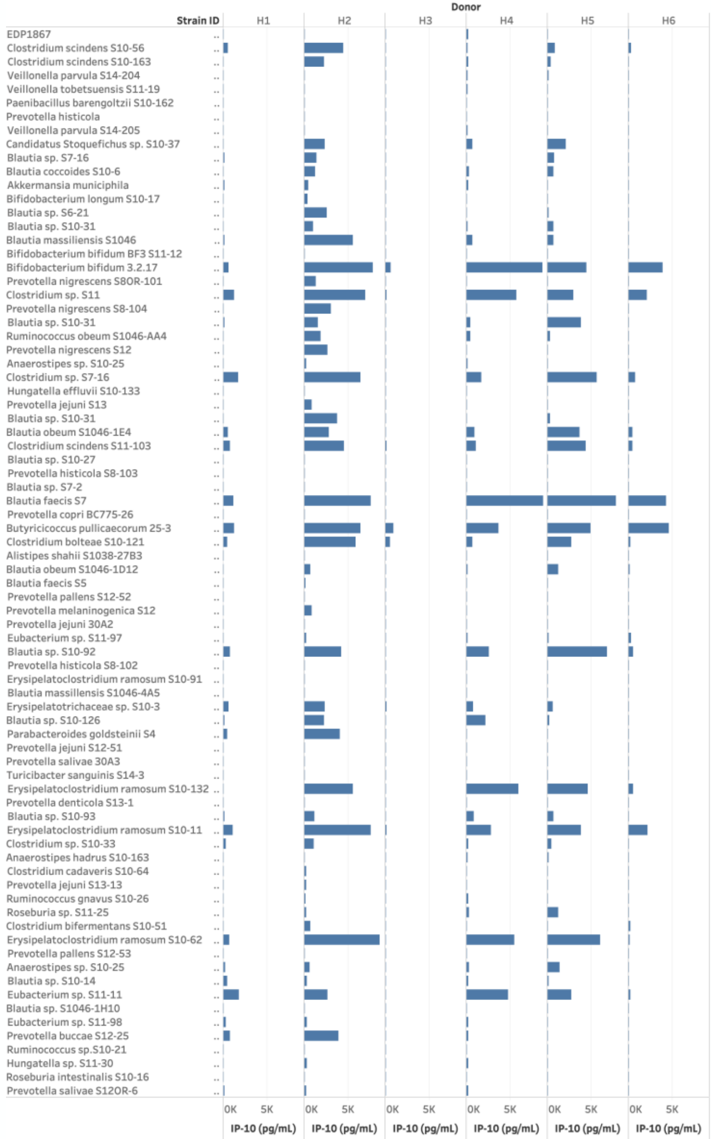

TNFa

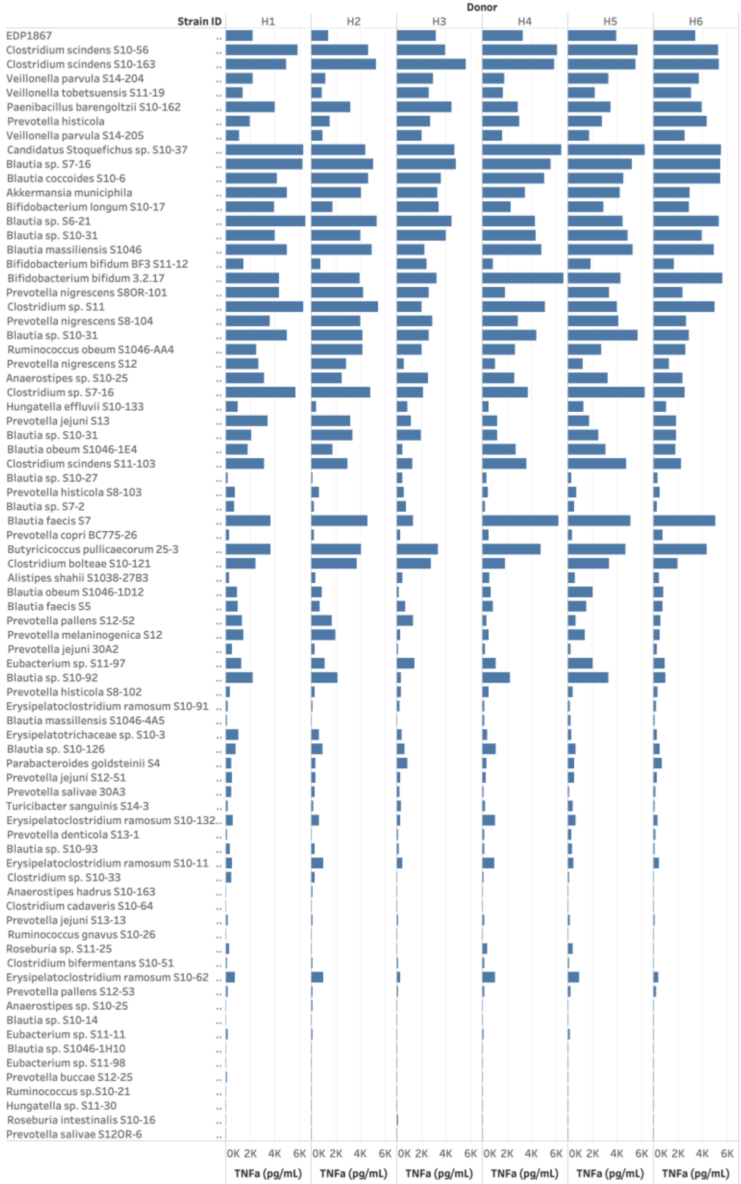

Human Macrophages were stimulated with different anaerobic bacterial strains for 24h and flushed with 1% oxygen and supernatants were collected to test for cytokine levels by MSD. Data shown from 6 independent human donors.

Supplementary Figure 2. EDP1867 is gut restricted and not detected in any other tissue

S2A

10 min

1 hr

6 hr

24 hr

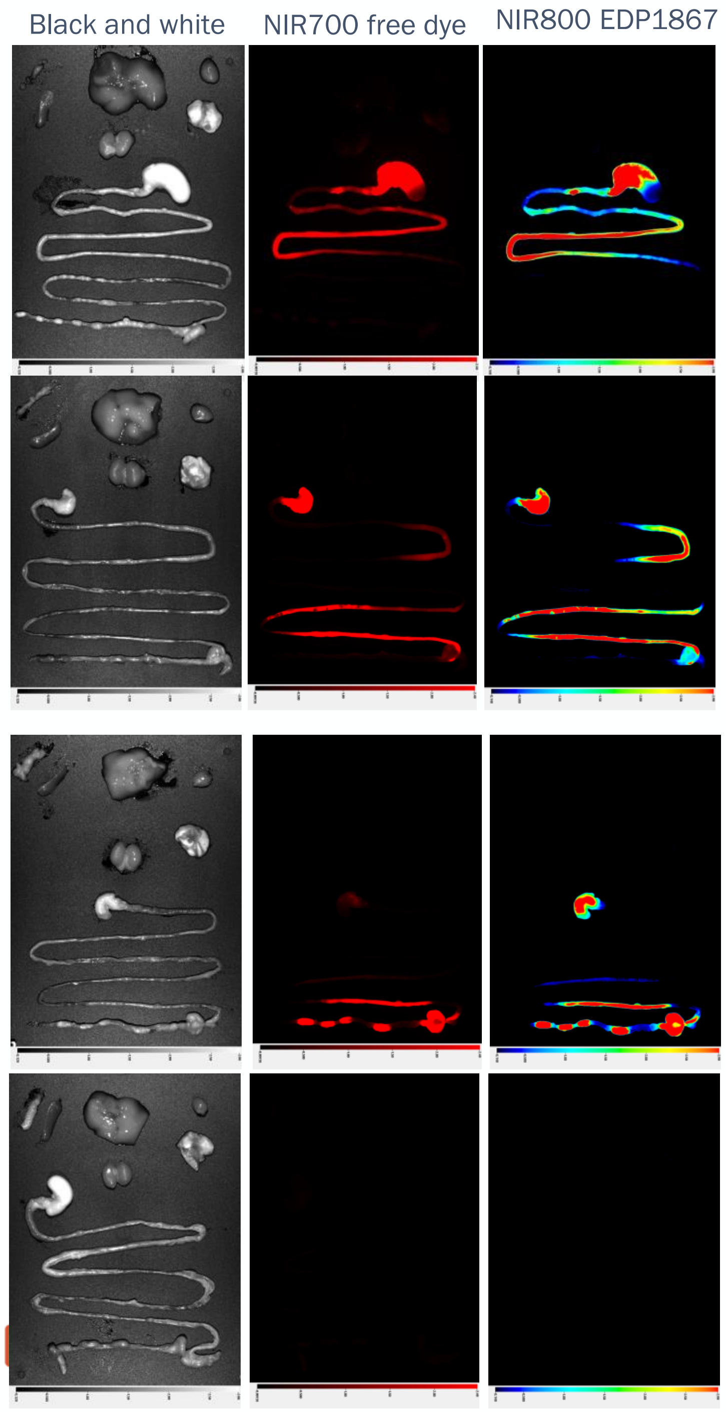

S2B

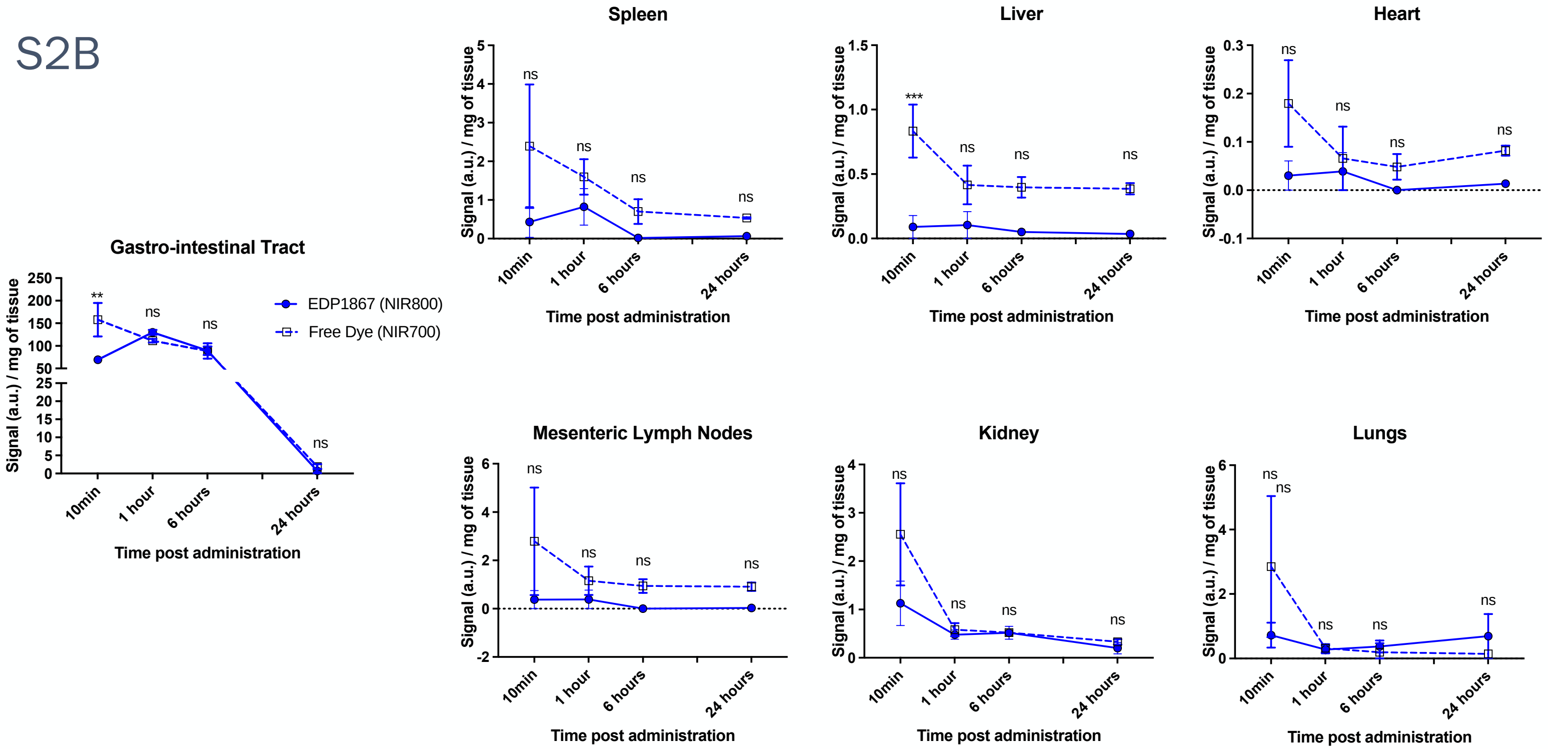

(A) Biodistribution of EDP1867. Following a single oral administration of EDP1867, no systemic exposure is observed at any timepoint in various organs and >99% of the total signal remains in the GIT. Left column-- brightfield images, middle- free dye, right- EDP1867 overlapped with free dye GIT- gastrointestinal tract (B) Signals for EDP1867 are minimal in systemic tissues such as spleen, liver, heart, mLN, kidney and lungs. EDP1867(solid blue line) tracks with baseline levels free dye control(dotted blue line). n= 3/mice/time point/group. Data are represented as mean+SEM. p values calculated and represented as **p <0.01, ***p< 0.001, ns > 0.1, as determined by 2-way ANOVA followed by Sidak's multiple comparison test

Supplementary Figure 3 - EDP1867 has no effects on microbiome

S3A

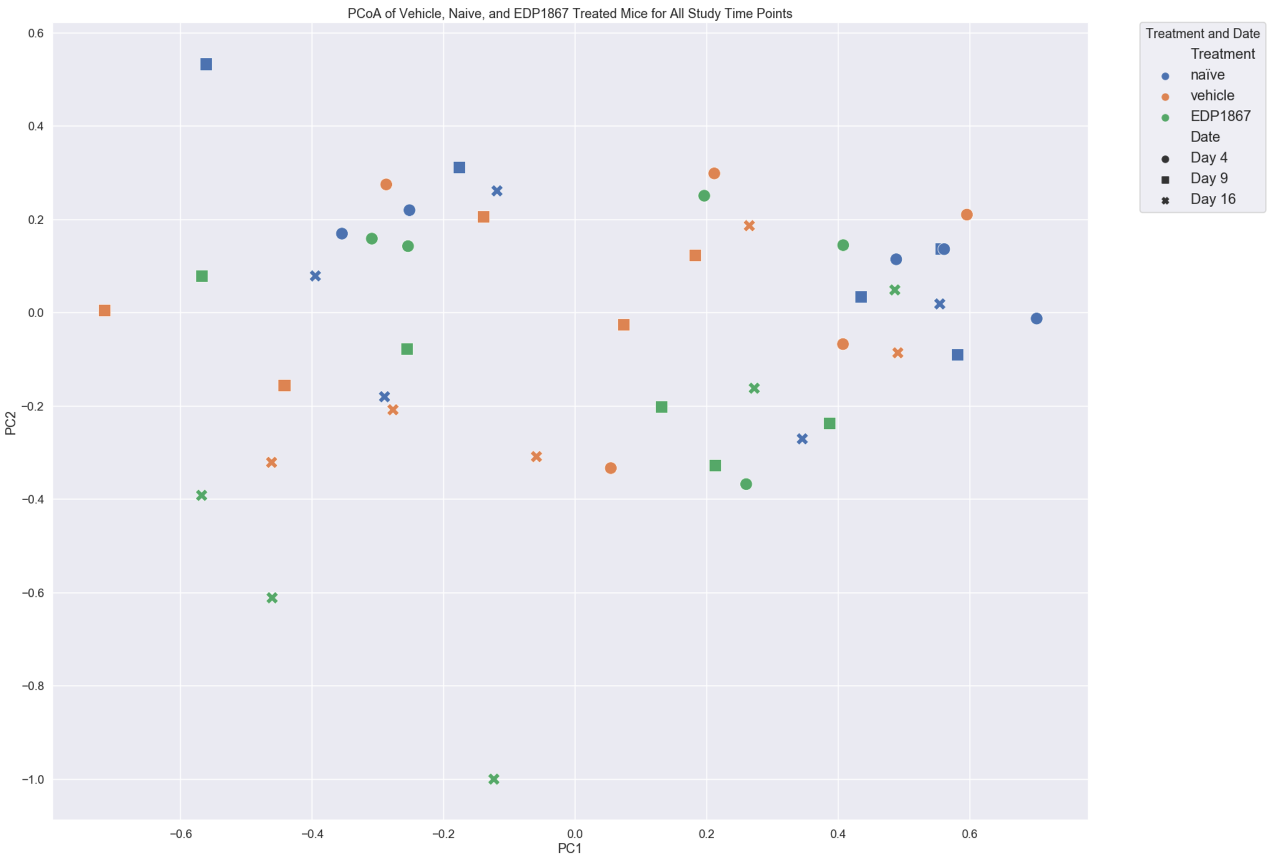

S3B

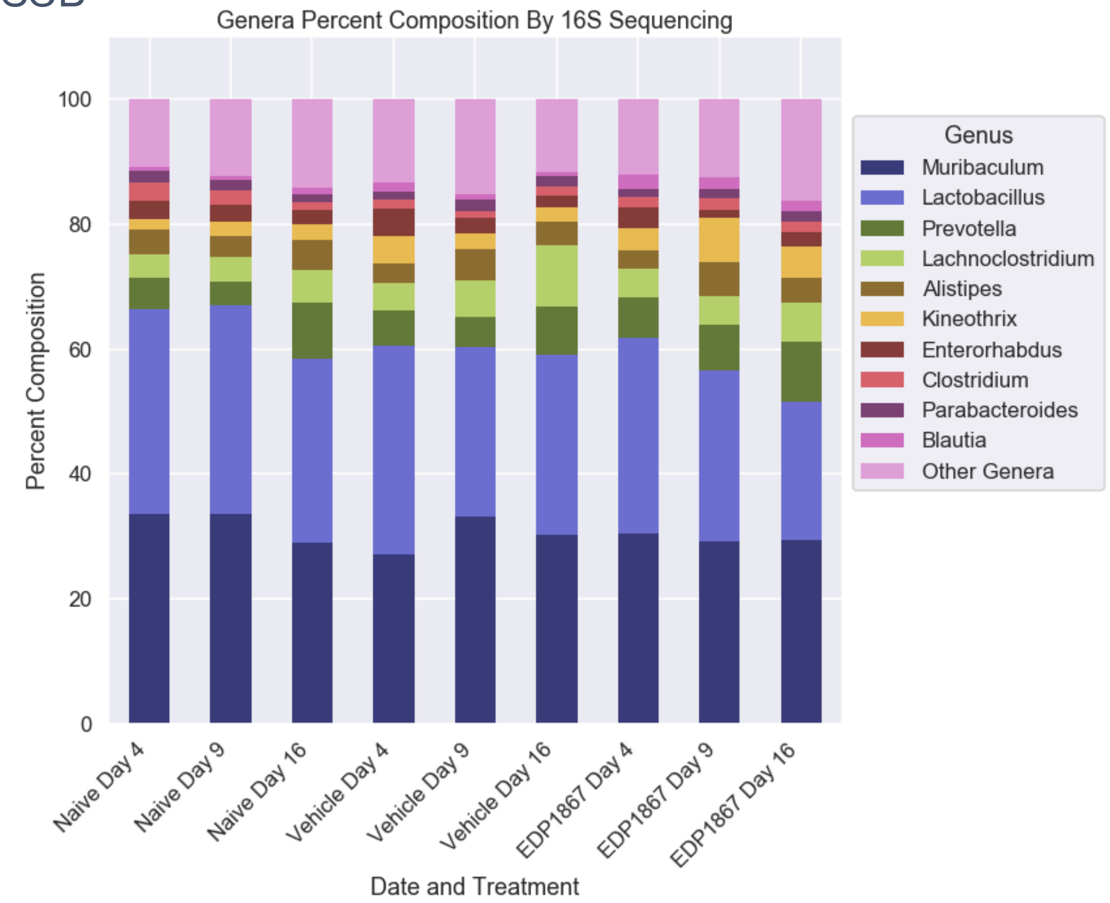

(A) Mean relative abundance of genera detected in the mouse fecal microbiome for each of the three treatment conditions at the day 4, day 9 and day 16 time points. (B) The 10 most abundant genera are listed by name, while the remaining are grouped. For all treatment conditions, the fecal microbiome composition is very stable across the three time points, and treatment with EDP1867 does not appear to cause a substantial shift in microbiome composition as measured by 16S sequencing.

Supplementary Figure 4. Therapeutic treatment with EDP1867 does not reduce inflammation of the spinal cord but reduces demyelination

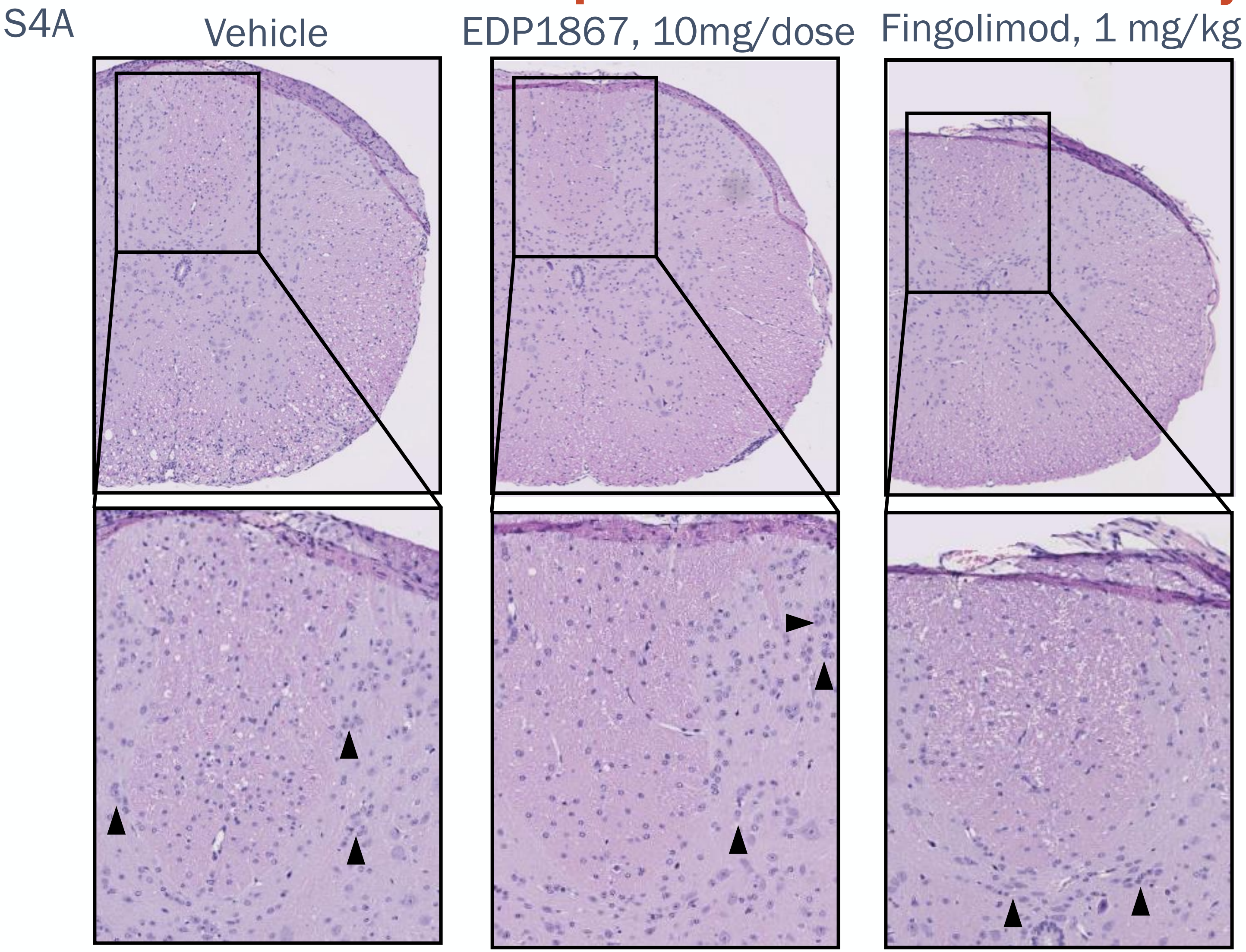

Images show cervical region of spinal cords
Arrows indicate accumulations of immune cell infiltration

(A) Representative hematoxylin and eosin (H&E)-stained images of the brain of spinal cords of mice treated with EDP1867, Vehicle or Fingolimod. Spinal cord sections are enlarged at 289X magnification to show regions with inflammation and inflammatory loci. Data are representative of 2 independent experiments (n=8 mice per group).

Supplementary Figure 4. Therapeutic treatment with EDP1867 does not reduce inflammation of the spinal cord but reduces demyelination

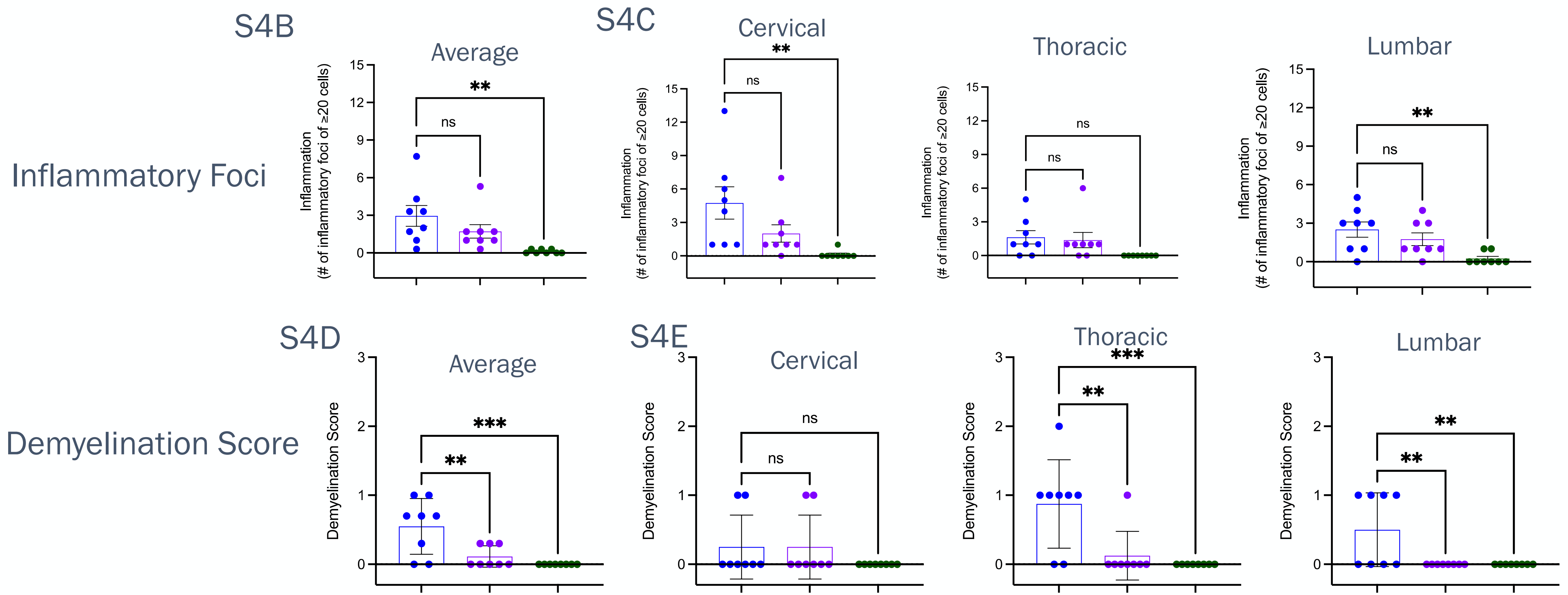

(C-D) Analysis of inflammatory foci of >20 cells in the spinal cords of mice treated with vehicle (blue), EDP1815 (purple), or Fingolimod 1mg/kg (dark green). C) Average number of inflammatory foci across all three regions of the spinal cord (cervical, lumbar, and thoracic). D) Quantification of inflammatory foci in each of the indicated regions of the spinal cord. E-F) Analysis of demyelination in the spinal cords of mice treated with vehicle (blue), EDP1815 (purple), or Fingolimod 1mg/kg (dark green) (n=8 mice per group). E) Average demyelination score across all three regions of the spinal cord (cervical, lumbar, and thoracic). F) Demyelination score of each of the indicated regions of the spinal cord. All data show mean ± SEM. **$p < 0.01$, ***$p < 0.001$, ns: not significant as determined by ordinary One-Way ANOVA

EVELO

Supplementary Figure 5. EAE RNASeq (duodenum vs colon) and duodenum immune cell subsets and pathway enrichment

S5A

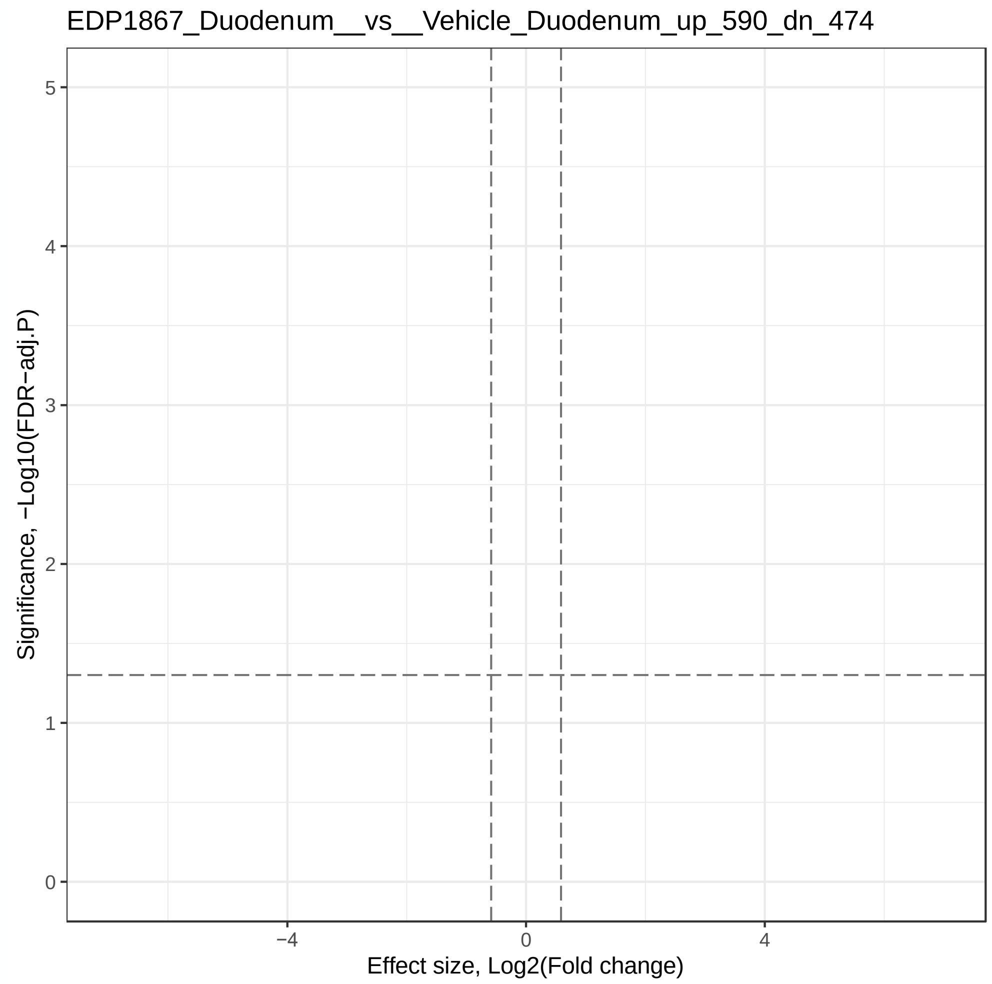

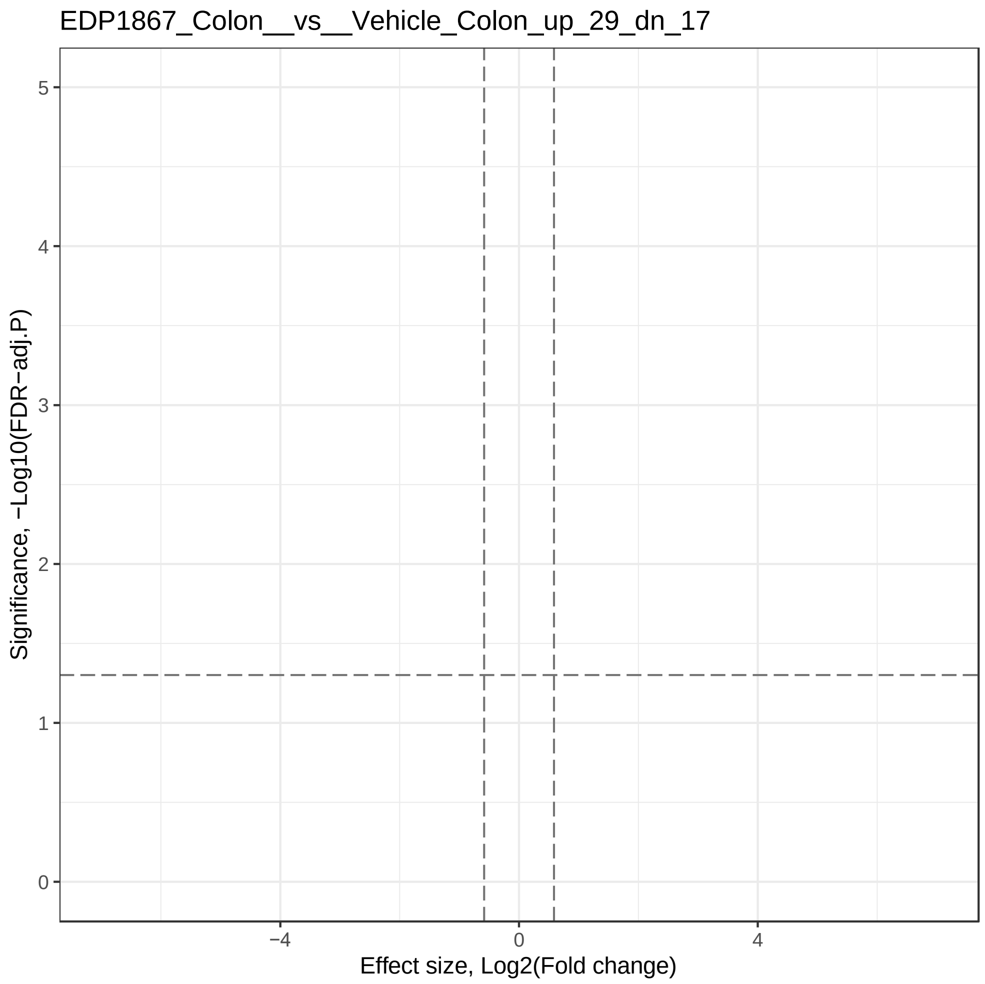

EVELO

Supplementary Figure 5. EAE RNASeq (duodenum vs colon) and duodenum immune cell subsets and pathway enrichment

S5B

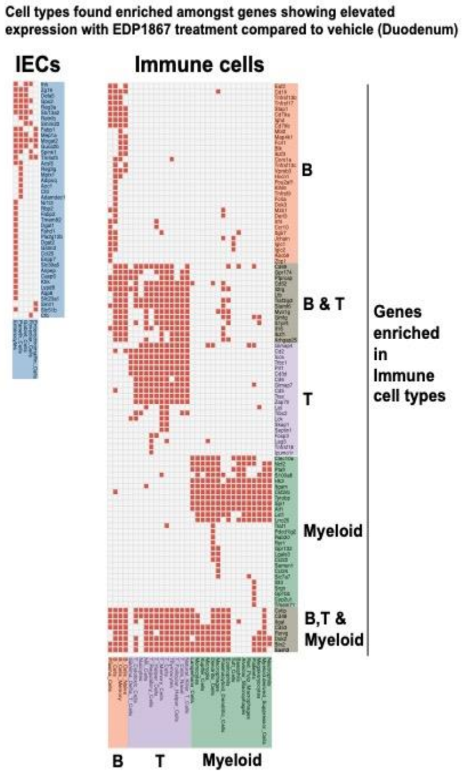

S5C

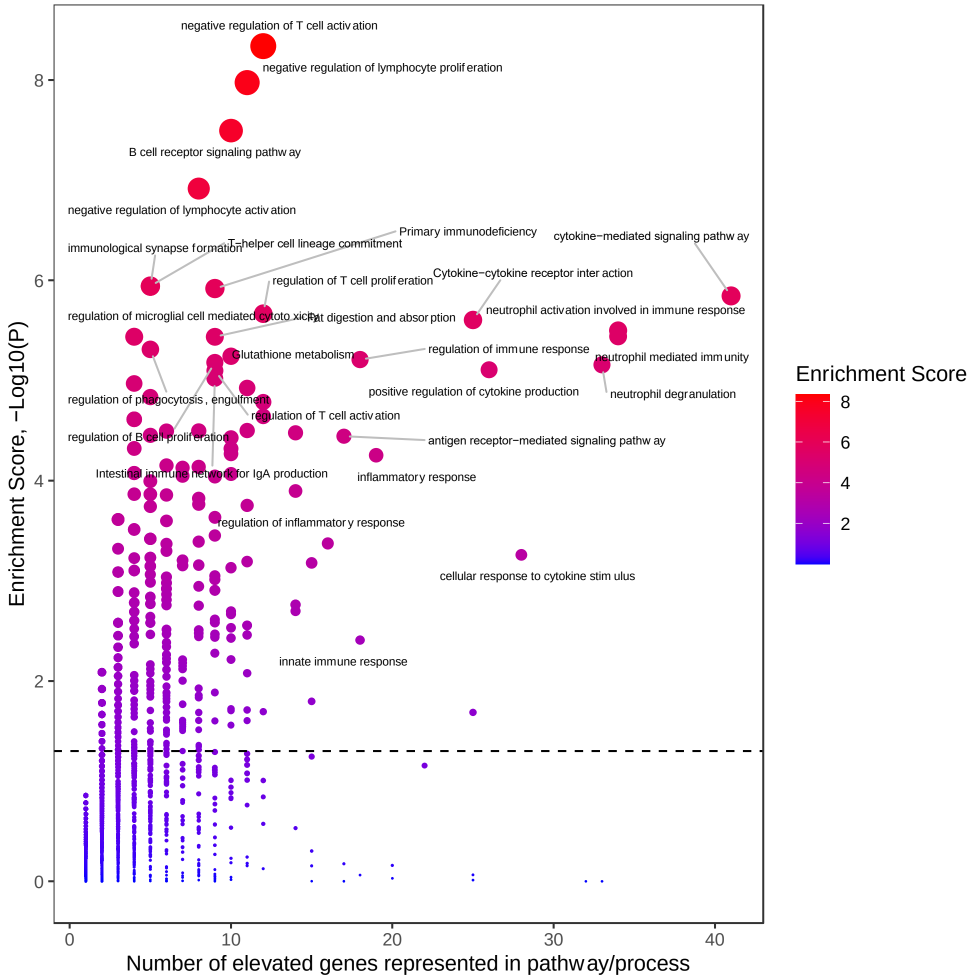

RNAseq analysis of mouse duodenum and colon treated with EDP1867 or vehicle control. (A)Violin plots showing differentially expressed genes triggered by EDP1867 compared to vehicle. In the duodenum, 590 genes showed elevated expression (Red) and 474 genes, a reduction in expression (Blue) due to EDP1867 treatment compared to vehicle. In the colon, EDP1867 treatment resulted in 29 genes with elevated expression and 17 genes, reduced expression relative to vehicle. These expression changes were at least 1.5-fold and were statistically significant (Benjamini-Hochberg-adjusted P-value ≤ 0.05). The other data points that did not meet these criteria were colored grey. (B)Cell type enrichment map of intestinal epithelial cells (IECs) and immune cell types showing cell subsets found enriched (P-value ≤ 0.05) amongst the 590 genes induced by EDP1867 treatment versus vehicle. (C)Pathway and biological processes enriched amongst EDP1867-induced genes. Size of data points and color (red) intensity scale with higher enrichment score. Dashed line denotes the threshold above which a pathway or process is significant (P-value ≤ 0.05).

Supplementary Figure 6 - EDP1867 is TLR2 dependent and TLR4 independent

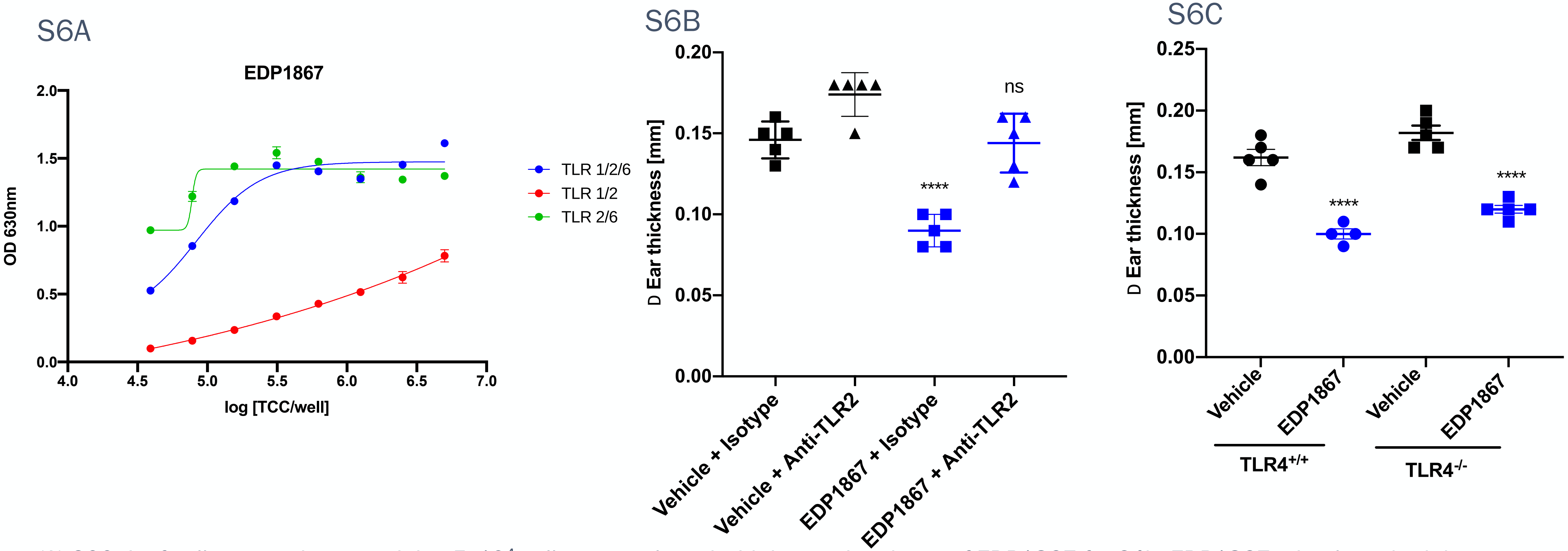

(A) 200uL of cell suspension containing $5x10^4$ cells were cultured with increasing doses of EDP1867 for 24h. EDP1867 stimulates both human TLR1/2 and TLR2/6 heterodimers, with greater potency observed for TLR2/6 heterodimer. KLH-DTH was induced as previously described. Mice were orally dosed with vehicle or EDP1867 (TCC- 2.16E+12) from day 5 through 8. Ear inflammation was measured on day 9. (A) Mice were treated with anti-TLR2 blocking antibody on days 2,4, and 6 as indicated and change in ear thickness was measured (C) Change in ear thickness (n = 5 mice/group) in C3HEJ (TLR4-deficient) and C3HEN (wildtype) mice dosed with EDP1867 (TCC- 7.8E+10) All studies have n = 5 mice/group. Representative figure from n = 1- 2 experiments. All data show mean ± SEM. **p < 0.01, ****p < 0.0001, ns: not significant as determined by ordinary One-Way ANOVA

Supplementary Figure 7. Validating of loss of migrating cells upon inhibition with combination of anti-LPAM-1 and anti–CD62L

S7A

Isotype control

Anti-LPAM-1/anti-CD62L

S7B

Isotype control

Anti-LPAM-1 /anti-CD62L

S7C

Isotype control

Anti-LPAM-1 /anti-CD62L

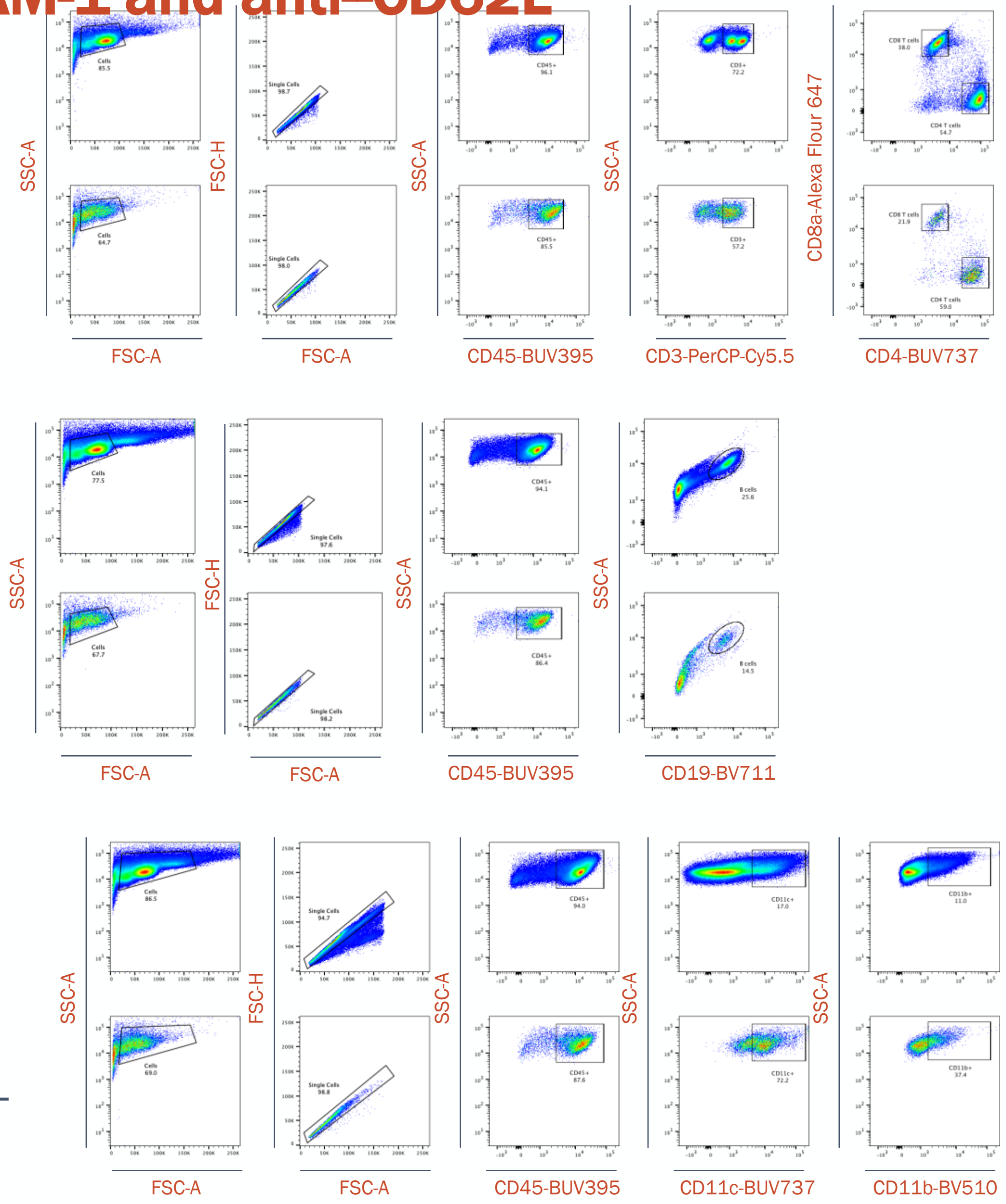

Flow cytometric analysis of mesenteric lymph node immune cell populations after inhibition of gut homing using a combination of anti-LPAM1 and anti-CD62L antibodies. Mice were treated with intraperitoneal injections of isotype control IgG2a or anti-LPAM1 and anti-CD62L antibodies every 2 days for 6 days. On day 7, mesenteric lymph nodes were harvested, homogenized, and assessed by flow cytometry. **A)** Gating strategy for T cells from either isotype control (upper row) or anti-LPAM1 and anti-CD62L treated mice. **B)** Gating strategy for B cells from either isotype control (upper row) or anti-LPAM1 and anti-CD62L treated mice. **C)** Gating strategy for myeloid cells from either isotype control (upper row) or anti-LPAM1 and anti-CD62L treated mice.

Supplementary Figure 7. Validating of loss of migrating cell upon inhibition with combination of anti-LPAM-1 and anti–CD62L

S7D

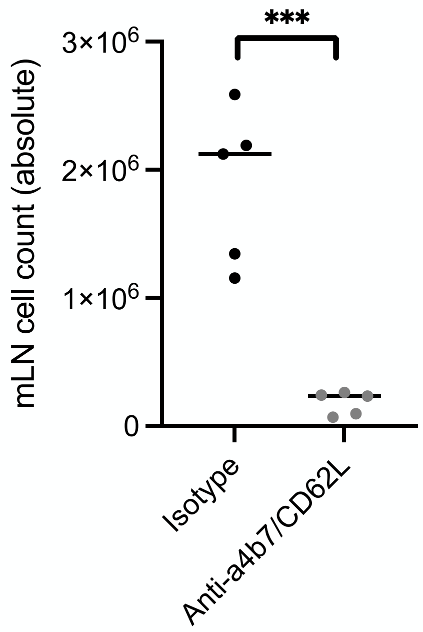

S7E

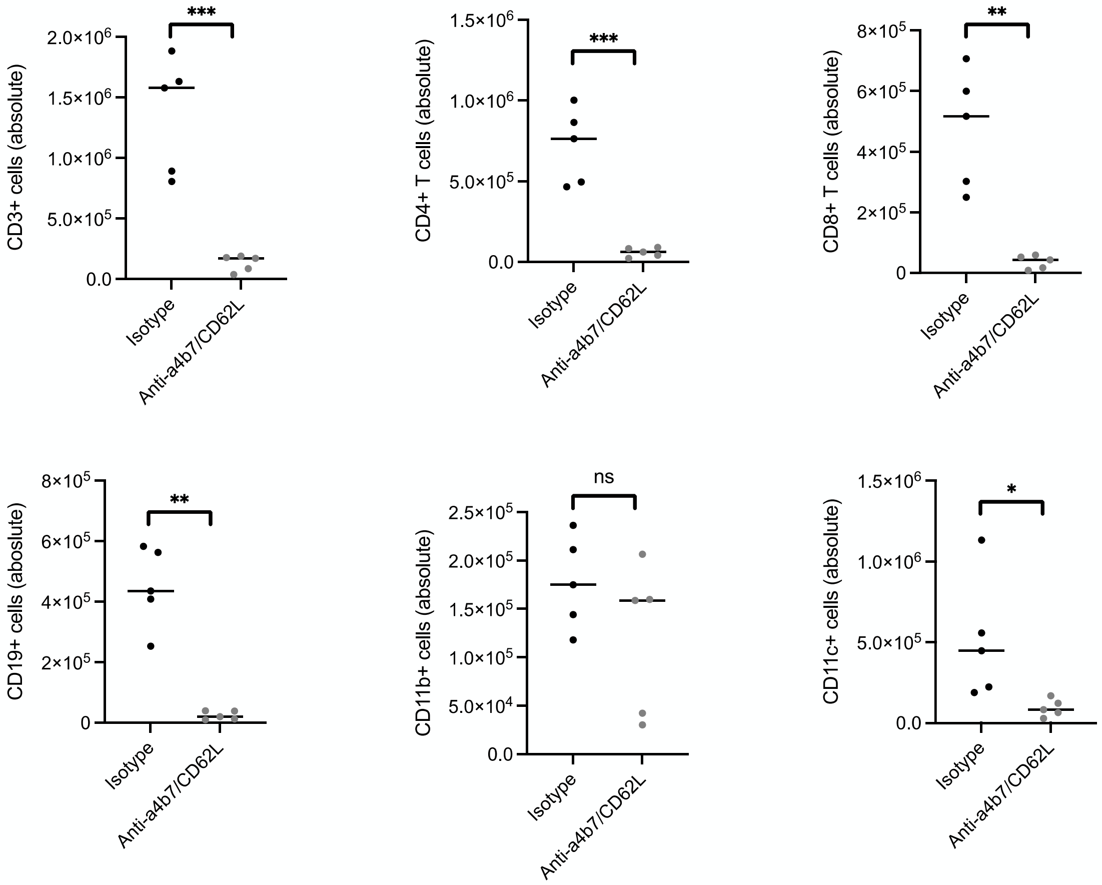

Mice were treated with intraperitoneal injections of isotype control IgG2a or anti-LPAM1 and anti-CD62L antibodies every 2 days for 6 days. On day 7, mesenteric lymph nodes were harvested, homogenized, cell number was counted, and then samples were assessed by flow cytometry. **D)** Graph shows the absolute numbers of cells in mesenteric lymph nodes from mice treated with isotype control or anti-LPAM1 and anti-CD62L treated mice. **E)** Graph show quantification of indicated cell populations in the mLN of mice treated with isotype control IgG2a or anti-LPAM1 and anti-CD62L antibodies. Statistical analysis was performed using a two-tailed unpaired Student’ t-test.
