# Supplementary material for: Regulation of Peripheral Inflammation by a Non-Viable, Non-Colonizing Strain of Commensal Bacteria
Source: Front Immunol. 2022 Feb 2;13:768076. doi: 10.3389/fimmu.2022.768076 (PMC8847375; doi:10.3389/fimmu.2022.768076)
Supplement: Supplementary file 2 [file DataSheet_2.docx]

Supplementary Material

# Supplementary Methods

***Human Inflamed Antigen Presenting Cell (APC) assay.***Freshly isolated PBMCs from 6 different human donors were used for isolation of CD14+ monocytes. PBMCs were washed in 10ml MACS buffer, spun down and resuspended at a concentration of 10^7^ total cells per 80μl. Anti-CD14+ beads were added (20μl per 10^7^ cells) and the cell suspension was incubated at 4^o^C for 15 minutes. Following incubation, cells were washed and resuspended in MACS buffer and CD14+ cells were isolated using magnetic separation as per manufacturer’s protocol (Miltenyi). Isolated CD14+ cells were further differentiated in flasks for 7 days at a concentration of 10^6^ cells/mL with the addition of 50ng/mL of gmCSF (every other day). On Day 8 APCs were harvested and inflamed with 10ng/mL of LPS + IFNg then 30,000 cells were plated per well in 100uL with fresh antibiotic free media. Microbes were added in anaerobic conditions and flushed with 1% oxygen. Plates were incubated for 24hrs in an anaerobic box at 37^o^C +5% CO2. After 24 hours, plates were centrifuged, and supernatants were collected to assay cytokine levels using Luminex assays.

***HEK-TLR assay.*** HEK293-SEAP reporter cells (Invivogen) expressing human TLR1, TLR2, and TLR6 combinations were plated at a final concentration of 20,000 cells per well in 96 well plates and cultured in appropriate selection media. After 48 hours selection media was washed out and replaced with complete media, and EDP1867 was added at the indicated concentrations per well. Cells were cultured in the presence of EDP1867 for 24 hours. Supernatant was collected and incubated with HEK-Blue reagent (Invivogen) for 1 hr, followed by reading absorbance at OD 630nm for SEAP production to determine stimulation of TLR2 heterodimers.

***RNA sequencing of small and large intestine*.** Mice were euthanized with CO_2_, then the first 4 cm of the small intestine and the colon were excised and placed in RNAlater (Sigma). Tissue samples were then incubated at 4ºC for 2 days, -20ºC for 2 days, and then stored at -80ºC until processing. Total RNA (from duodenum tissue taken on day 42) was extracted using the RNeasy Mini Kit (Qiagen) and quantified using the Qubit 2.0 Fluorometer (Life Technologies, CA). RNA integrity was checked using Agilent TapeStation 4200 (Agilent Technologies, CA). RNA-seq libraries were prepared using the NEBNext Kit (New England Biolabs, MA) according to manufacturer’s instructions. Samples were sequenced to a target depth of 20 million paired-end reads with Illumina 2x150-base pair technology. Sequence reads were mapped using the STAR aligner (Dobin et al., 2013) and quantified using RSEM(Li and Dewey, 2011). Data was normalized using trimmed mean of M-values (TMM), followed by variance estimation, and applying generalized linear models (GLMs), utilizing functions from empirical analysis of digital gene expression to identify differentially expressed genes (McCarthy et al., 2012). P-values were adjusted to control false discovery rate (FDR) in multiple testing using the Benjamini and Hochberg method. Differential gene expression was defined as a fold change of ≥1.5 and FDR-adj. p≤0.05. Enrichment and over-representation analyses were performed as previously described (Bouziat et al., 2017; Varma et al., 2020) using datasets and gene sets from publicly available sources including KEGG (Kanehisa et al., 2017), Gene Ontology (Gene Ontology, 2021), GTEx (Consortium, 2013), MSigDB (Liberzon et al., 2015), ImmGen (Immunological Genome, 2020), Human Cell Atlas (Franzen et al., 2019)and other single-cell data collections from EMBL-EBI (Papatheodorou et al., 2020), Broad Institute (Broad Institute’s Single-Cell Portal: https://singlecell.broadinstitute.org/single_cell), RIKEN (Abugessaisa et al., 2018), Karolinska Institute(Franzen et al., 2019) and University of Texas Health (Yuan et al., 2021 bioRxiv 104810; doi: https://doi.org/10.1101/104810).

***Flow Cytometry*.** Mice were euthanized with CO_2_, then the mesenteric lymph nodes were excited, homogenized through a 70um cell strainer, and washed with FACS Buffer (4% BFS in PBS) at 300 x g for 5 mins at 4ºC. Cells were then resuspended in TruStain FcX anti-mouse CD16/32 (1:100 in FACS Buffer; BioLegend) and incubated at 4ºC for 15 mins. Cells were then washed with FACS Buffer (300 x g, 5 mins, 4ºC) and resuspended in T cell, B cell, or myeloid cell staining cocktails (see antibody section). Samples were then incubated at 4ºC for 30 mins, washed with FACS Buffer (300 x g, 5 mins, 4ºC), then resuspended in 200 uL FACS Buffer. Samples were then analyzed by flow cytometry (BD Fortessa; BD Biosciences) and populations were assessed using FloJo v10 (BD Biosciences).

***Antibodies and Flow Cytometry Panels*.** Antibodies used for flow cytometry were CD45-BUV 395 Clone 30-F11, CD4-BUV737 Clone RM4-5, CD11c BUV737 Clone HL3 (all BD Biosciences), CD8a-AlexaFluor647 Clone 53-6.7, CD19-BV711 Clone 6D5, and CD11b-BV510 Clone M1/70 (all BioLegend). Flow cytometry panels were the follow: T cells were analyzed using CD45, CD3, CD4, and CD8a; B cells were analyzed using CD45 and CD19; myeloid cells were analyzed using CD45, CD11b, and CD11c.

**References**

Abugessaisa, I., Noguchi, S., Bottcher, M., Hasegawa, A., Kouno, T., Kato, S., Tada, Y., Ura, H., Abe, K., Shin, J.W.*, et al.* (2018). SCPortalen: human and mouse single-cell centric database. Nucleic Acids Res *46*, D781-D787.

Bouziat, R., Hinterleitner, R., Brown, J.J., Stencel-Baerenwald, J.E., Ikizler, M., Mayassi, T., Meisel, M., Kim, S.M., Discepolo, V., Pruijssers, A.J.*, et al.* (2017). Reovirus infection triggers inflammatory responses to dietary antigens and development of celiac disease. Science *356*, 44-50.

Consortium, G.T. (2013). The Genotype-Tissue Expression (GTEx) project. Nat Genet *45*, 580-585.

Dobin, A., Davis, C.A., Schlesinger, F., Drenkow, J., Zaleski, C., Jha, S., Batut, P., Chaisson, M., and Gingeras, T.R. (2013). STAR: ultrafast universal RNA-seq aligner. Bioinformatics *29*, 15-21.

Franzen, O., Gan, L.M., and Bjorkegren, J.L.M. (2019). PanglaoDB: a web server for exploration of mouse and human single-cell RNA sequencing data. Database (Oxford) *2019*.

Gene Ontology, C. (2021). The Gene Ontology resource: enriching a GOld mine. Nucleic Acids Res *49*, D325-D334.

Immunological Genome, P. (2020). ImmGen at 15. Nat Immunol *21*, 700-703.

Kanehisa, M., Furumichi, M., Tanabe, M., Sato, Y., and Morishima, K. (2017). KEGG: new perspectives on genomes, pathways, diseases and drugs. Nucleic Acids Res *45*, D353-D361.

Li, B., and Dewey, C.N. (2011). RSEM: accurate transcript quantification from RNA-Seq data with or without a reference genome. BMC Bioinformatics *12*, 323.

Liberzon, A., Birger, C., Thorvaldsdottir, H., Ghandi, M., Mesirov, J.P., and Tamayo, P. (2015). The Molecular Signatures Database (MSigDB) hallmark gene set collection. Cell Syst *1*, 417-425.

McCarthy, D.J., Chen, Y., and Smyth, G.K. (2012). Differential expression analysis of multifactor RNA-Seq experiments with respect to biological variation. Nucleic Acids Res *40*, 4288-4297.

Papatheodorou, I., Moreno, P., Manning, J., Fuentes, A.M., George, N., Fexova, S., Fonseca, N.A., Fullgrabe, A., Green, M., Huang, N.*, et al.* (2020). Expression Atlas update: from tissues to single cells. Nucleic Acids Res *48*, D77-D83.

Varma, M., Kadoki, M., Lefkovith, A., Conway, K.L., Gao, K., Mohanan, V., Tusi, B.K., Graham, D.B., Latorre, I.J., Tolonen, A.C.*, et al.* (2020). Cell Type- and Stimulation-Dependent Transcriptional Programs Regulated by Atg16L1 and Its Crohn's Disease Risk Variant T300A. J Immunol *205*, 414-424.
